# Fibroblast-specific IKK-**β** deficiency ameliorates angiotensin II–induced adverse cardiac remodeling in mice

**DOI:** 10.1172/jci.insight.150161

**Published:** 2021-09-22

**Authors:** Weiwei Lu, Zhaojie Meng, Rebecca Hernandez, Changcheng Zhou

**Affiliations:** 1 Department of Pharmacology and Nutritional Sciences, College of Medicine, University of Kentucky, Lexington, Kentucky, USA.; 2 Division of Biomedical Sciences, School of Medicine, University of California, Riverside, California, USA.

**Keywords:** Cardiology, Cardiovascular disease, Mouse models, NF-kappaB

## Abstract

Cardiac inflammation and fibrosis contribute significantly to hypertension-related adverse cardiac remodeling. IκB kinase β (IKK-β), a central coordinator of inflammation through activation of NF-κB, has been demonstrated as a key molecular link between inflammation and cardiovascular disease. However, the cell-specific contribution of IKK-β signaling toward adverse cardiac remodeling remains elusive. Cardiac fibroblasts are one of the most populous nonmyocyte cell types in the heart that play a key role in mediating cardiac fibrosis and remodeling. To investigate the function of fibroblast IKK-β, we generated inducible fibroblast-specific IKK-β–deficient mice. Here, we report an important role of IKK-β in the regulation of fibroblast functions and cardiac remodeling. Fibroblast-specific IKK-β–deficient male mice were protected from angiotensin II–induced cardiac hypertrophy, fibrosis, and macrophage infiltration. Ablation of fibroblast IKK-β inhibited angiotensin II–stimulated fibroblast proinflammatory and profibrogenic responses, leading to ameliorated cardiac remodeling and improved cardiac function in IKK-β–deficient mice. Findings from this study establish fibroblast IKK-β as a key factor regulating cardiac fibrosis and function in hypertension-related cardiac remodeling.

## Introduction

Cardiac remodeling is a major process responsible for end-stage heart failure, a leading cause of morbidity and mortality worldwide (1–3). Left ventricular (LV) hypertrophy, cardiac inflammation, and fibrosis are the key aspects of hypertension-related cardiac remodeling, which can be caused by hemodynamic load, neurohumoral activation, growth factors, and cytokines (4). Among the neurohumoral factors, angiotensin II (Ang II), a key component of the renin-angiotensin system, plays an important role in the pathogenesis of cardiac remodeling by inducing hypertension and inflammation in a variety of cardiac diseases (5). It has been demonstrated that cardiac inflammation and recruitment of immune cells significantly contribute to adverse cardiac remodeling in the pathophysiology of heart failure in animal and human studies (6, 7). Ang II has been shown to activate a number of signaling pathways, including NF-κB, mitogen-activated protein kinases, and ROS to induce cardiac hypertrophy, inflammation, and fibrosis (8).

Many inflammatory signaling pathways that contribute to cardiovascular disease are regulated by the transcriptional factor NF-κB, a master regulator of the innate and adaptive immune responses (9–11). IκB kinase β (IKK-β) is the predominant catalytic subunit of the IKK complex and is required for activation of NF-κB by inflammatory mediators in the canonical or classical activation pathway (9, 11, 12). We and others have recently revealed the important function of IKK-β in cardiovascular disease and metabolic disorders (11, 13–18). For example, we found that deficiency of myeloid IKK-β reduced macrophage inflammatory responses and decreased diet-induced atherosclerosis in hyperlipidemic, LDL receptor-deficient (LDLR^–/–^) mice (13). Deletion of IKK-β in smooth muscle cells protected LDLR^–/–^ mice from diet-induced vascular inflammation and atherosclerosis (11). We also found that many adipocyte precursor cells express smooth muscle cell markers, and ablation of IKK-β in those cells inhibits adipocyte differentiation and renders mice resistant to diet-induced obesity (11, 16, 19). Interestingly, targeted deletion of IKK-β in mature adipocytes affects adipose remodeling, tissue inflammation, and atherosclerotic plaque vulnerability in obese LDLR^–/–^ mice (20).

In addition to vascular diseases, IKK-β/NF-κB signaling has also been implicated in cardiac pathogenesis, such as cardiac hypertrophy (21, 22), ischemia/reperfusion damage (23), and myocardial infarction (24, 25). For instance, overexpression of an active form of IKK-β in cardiomyocytes led to myocarditis, inflammatory dilated cardiomyopathy, and muscle fiber atrophy in mice (26). Consistently, inhibition of IKK-β attenuated the excessive inflammation and systolic cardiac dysfunction associated with sepsis in patients with chronic kidney disease (27). Paradoxically, Hikoso et al. found that ablation of IKK-β in cardiomyocytes increased pressure overload–induced cardiomyocyte apoptosis, leading to more serious cardiac dysfunction and chamber dilation compared with control littermates (28). These findings suggest that the functions of the IKK-β pathway in cardiac disease are complex, and further studies are required to define the cell/tissue-specific role of IKK-β in hypertensive cardiac remodeling.

Cardiomyocytes have been widely studied for their role in the pathogenesis of cardiac disease, but there is increasing evidence that cardiac fibroblasts, one of the most populous nonmyocyte cell types in the heart, also play an important role in mediating cardiac fibrosis and remodeling (7, 29). Fibroblasts can transdifferentiate into pathologically activated myofibroblasts, which secrete excessive amounts of matrix molecules and therefore lead to accumulation of cardiac collagen (30). In addition, activated fibroblasts have elevated inflammatory responses that initiate the vicious circle of cardiac inflammation (31–33). Although several studies found that modulation of IKK-β activation or expression can affect myofibroblast formation and collagen synthesis in vitro (34, 35), the functions of fibroblast IKK-β in regulating cardiac function and remodeling in vivo remain poorly understood. In the present study, we generated inducible fibroblast-specific IKK-β–deficient mice and demonstrated that fibroblast IKK-β plays a key role in mediating Ang II–induced adverse cardiac remodeling and cardiac dysfunction in male mice.

## Results

### IKK-β mediates Ang II–induced cardiac fibroblast proinflammatory and profibrogenic responses in vitro.

**Figure 1:**
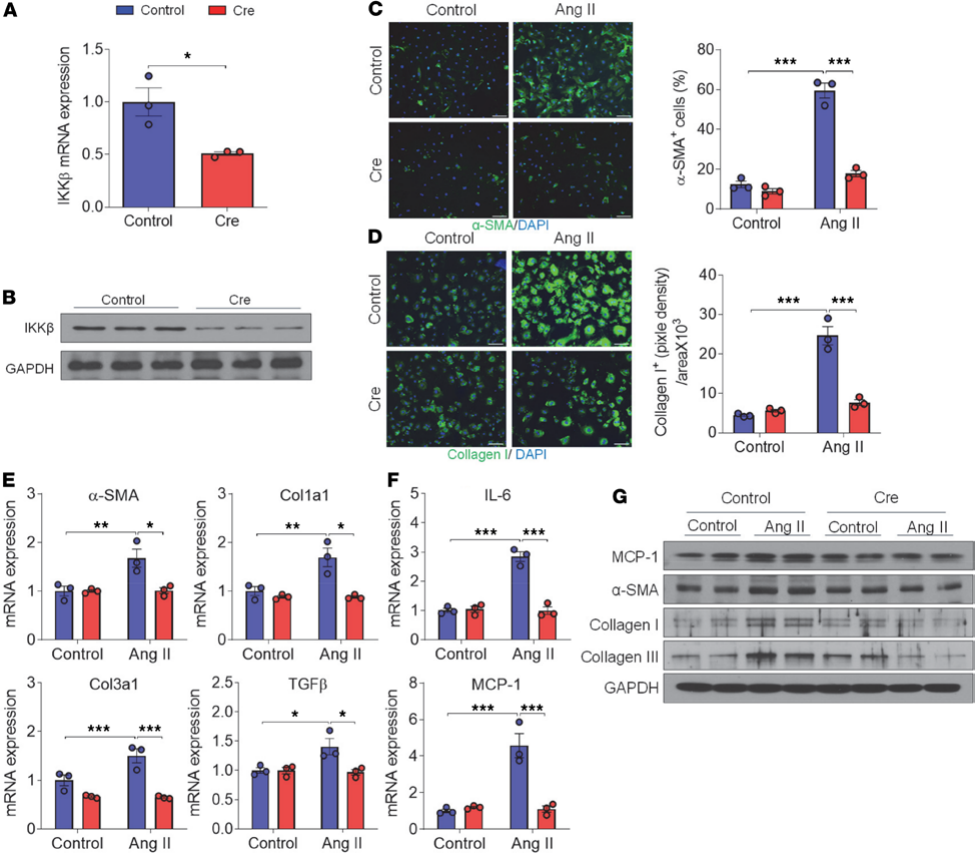
Knockdown of IKK-β inhibits angiotensin II–induced cardiac fibroblast proinflammatory and profibrogenic responses in vitro. (**A** and **B**) Primary cardiac fibroblasts (CFs) were isolated from 10-week-old male IKKβ^fl/fl^ mice and were infected with control (β-gal) or Cre lentivirus. IKK-β mRNA (**A**) or protein (**B**) levels were analyzed by qPCR or Western blot 4 days after the infection. (*n =* 3, Student’s *t* test, *P < 0.05). (**C**–**G**) CFs were treated with vehicle or 10^–6^ M of angiotensin II (Ang) II for 24 hours. Representative images of immunofluorescence staining (left) and the quantitation (right) of smooth muscle α-actin–positive (αSMA^+^) cells (**C**) and collagen I (**D**) in CFs. α-SMA^+^ stress fibers and collagen I are shown in green. The nuclei were visualized with DAPI (blue) (*n =* 3; 2-way ANOVA; ***P < 0.001; scale bar: 100 μm). qPCR analysis of the mRNA levels of fibrotic genes and inflammatory genes (**E**) (*n =* 3; 2-way ANOVA; *P < 0.05, **P < 0.01, ***P < 0.001.). Western blot analysis of the protein levels of MCP-1, α-SMA, collagen I, and collagen III (**G**).

To investigate the role of IKK-β in regulating cardiac fibroblast inflammation and fibrosis, primary cardiac fibroblasts were isolated from mice carrying loxP-flanked IKK-β alleles (IKKβ^fl/fl^) and then infected with lentivirus expressing Cre recombinase. The mRNA and protein levels of IKK-β were significantly decreased in cardiac fibroblasts infected with lentivirus expressing Cre as compared with those transduced with control lentivirus (Figure 1, A and B). Activation of fibroblasts to myofibroblasts is a key event in cardiac remodeling, and myofibroblasts are the dominant source of collagen, which contributes to this process.

Next, control and IKK-β–knockdown cardiac fibroblasts were stimulated with Ang II, and the differentiation of fibroblasts into myofibroblasts was evaluated. As shown in Figure 1C, Ang II treatment induced approximately 60%–70% of control fibroblasts differentiating to myofibroblasts, which expressed myofibroblast marker α-SMA. Cre-mediated IKK-β knockdown attenuated Ang II–induced fibroblast differentiation. Increased production and deposition of collagen type I by myofibroblasts is one of the main features of fibrosis (36). Consistently, immunofluorescence staining results also showed elevated collagen I protein levels in control fibroblasts in response to Ang II treatment, and the induction was reduced in IKK-β–knockdown fibroblasts (Figure 1D). Quantitative PCR (qPCR) and immunoblotting analyses then confirmed that Ang II treatment stimulated the expression of fibrotic markers, including αSMA, collagen 1a1 (Col1a1), and collagen 3a1 (Col3a1), in control but not IKK-β–knockdown fibroblasts (Figure 1, E and G). In addition, we found that the mRNA levels of TGF-β, a key mediator of myofibroblast transition (37), were increased by Ang II treatment in control but not IKK-β–knockdown fibroblasts (Figure 1E). Further, Ang II exposure also increased the expression of key inflammatory genes, IL-6 and monocyte chemotactic protein-1 (MCP-1), in control fibroblasts, but knockdown of IKK-β attenuated the elevated proinflammatory gene expression (Figure 1, F and G).

In addition to the Cre-mediated deletion approach, a highly selective IKK-β inhibitor, BMS-345541 (11, 14), was also used as a pharmacological approach. BMS-345541 treatment efficiently inhibited phosphorylation of IKK-β and p65 elicited by Ang II treatment in cardiac fibroblasts (Supplemental Figure 1A; supplemental material available online with this article; https://doi.org/10.1172/jci.insight.150161DS1). Consistent with IKK-β knockdown results (Figure 1), BMS-345541–mediated IKK-β inhibition attenuated Ang II–stimulated expression of fibroblast proinflammatory genes and fibrotic markers (Supplemental Figure 1B). BMS-345541 treatment decreased Ang II–induced fibroblast differentiation and collagen I production (Supplemental Figure 1C and 1D). Taken together, these in vitro results suggest a potential role of IKK-β signaling in mediating Ang II–induced inflammatory and fibrogenic responses.

*Generation of inducible fibroblast-specific IKK-β–deficient mice*. To further investigate the role of fibroblast IKK-β in regulating cardiac inflammation, fibrosis, and function in vivo, inducible fibroblast-specific IKK-β–deficient mice (termed IKKβ^ΔFib^) were generated by crossing mice carrying loxP-ﬂanked IKK-β alleles (IKKβ^fl/fl^; refs. 11, 19) with transgenic mice expressing tamoxifen-inducible Cre recombinase driven by the mouse collagen type I alpha 2 (Col1a2) promoter, a widely used model for in vivo analysis of gene function in fibroblasts as previously described (38, 39). Eight-week-old male IKKβ^ΔFib^ mice were i.p. injected with 2 mg tamoxifen per day for 5 days to induce Cre expression (40). IKKβ^ΔFib^ mice were viable and remained healthy and phenotypically normal after tamoxifen treatment (data not shown).

**Figure 2:**
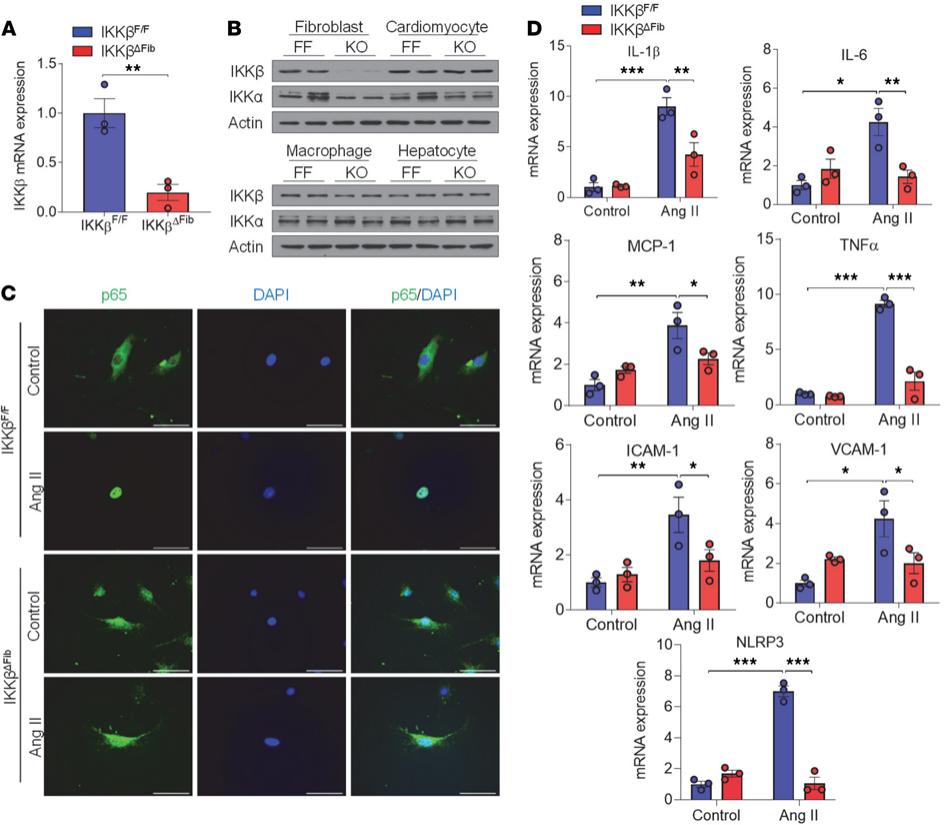
Generation of inducible fibroblast-specific IKK-β–deficient mice. (**A**) Eight-week-old male fibroblast-specific IKK-β–knockout mice (termed IKKβ^ΔFib^) and their male littermate controls (IKKβ^fl/fl^) were i.p. injected with 2 mg tamoxifen per day for 5 days to induce Cre expression. Cardiac fibroblasts (CFs) were isolated from IKKβ^fl/fl^ and IKKβ^ΔFib^ mice 1 week after the completion of tamoxifen dosing (to allow the clearance of tamoxifen). qPCR analysis of IKK-β mRNA levels in CFs (*n =* 3, Student’s *t* test, **P < 0.01). (**B**) Western blot analysis of IKK-β and IKK-α proteins in CFs, cardiomyocytes, bone marrow macrophages, and hepatocytes isolated from IKKβ^fl/fl^ and IKKβ^ΔFib^ mice. (**C**) Representative images of immunofluorescence staining of NF-κB p65 subunit (green) in CFs of IKKβ^fl/fl^ and IKKβ^ΔFib^ mice stimulated with 10^–6^ M of angiotensin II (Ang) II or vehicle control for 3 hours. The nuclei were visualized with DAPI (blue) (scale bar: 100 μm). (**D**) qPCR analysis of the mRNA levels of proinflammatory genes and adhesion molecules in CFs of IKKβ^fl/fl^ and IKKβ^ΔFib^ mice stimulated with 10^–6^ M of Ang II or vehicle control for 18 hours (*n =* 3; 2-way ANOVA; *P < 0.05, **P < 0.01, ***P < 0.001).

Next, cardiac fibroblasts were isolated from male IKKβ^fl/fl^ and IKKβ^ΔFib^ littermates and qPCR analysis was performed. The results confirmed the significantly decreased IKK-β mRNA levels in fibroblasts of IKKβ^ΔFib^ mice as compared with IKKβ^fl/fl^ control littermates (Figure 2A). Further, immunoblotting analysis also demonstrated that the protein levels of IKK-β were reduced in cardiac fibroblasts but not in other cell types, including cardiomyocytes, bone marrow macrophages, and hepatocytes of IKKβ^ΔFib^ mice as compared with IKKβ^fl/fl^ mice (Figure 2B). In addition, the protein levels of IKK-α were not affected in cardiac fibroblasts or other cell types of IKKβ^ΔFib^ mice. These results indicate specific and efficient IKK-β deletion in the cardiac fibroblasts of IKKβ^ΔFib^ mice.

To investigate whether deficiency of IKK-β affects cardiac fibroblast inflammatory responses, cardiac fibroblasts isolated from male IKKβ^fl/fl^ and IKKβ^ΔFib^ mice were treated with NF-κB stimulators, LPS, and Ang II. Consistent with our previous studies in other cell types (11, 13), treatment with those stimulators stimulated NF-κB subunit p65 translocation from the cytoplasm to the nucleus of control fibroblasts of IKKβ^fl/fl^ mice, but deficiency of IKK-β inhibited LPS- or Ang II–induced p65 translocation (Figure 2C and Supplemental Figure 2A). Consistently, gene expression analyses also demonstrated that the ability of Ang II to induce expression of mRNAs encoding IL-6, MCP-1, TNF-α, IL-1β, ICAM-1, and VCAM-1 was abrogated in cardiac fibroblasts of IKKβ^ΔFib^ mice (Figure 2D and Supplemental Figure 2B). Interestingly, deficiency of IKK-β also decreased Ang II–induced expression of another NF-κB target gene, NLRP3, in cardiac fibroblasts (Figure 2D). These results suggest that ablation of IKK-β attenuated NF-κB activity and reduced NF-κB–regulated gene expression of proinﬂammatory factors and adhesion molecules in cardiac fibroblasts.

*Deficiency of fibroblast IKK-βprotects mice from Ang II–induced adverse cardiac remodeling and cardiac dysfunction*. We next sought to determine whether activation of IKK-β signaling in cardiac fibroblasts is associated with cardiac remodeling and dysfunction. Ten-week-old male IKKβ^fl/fl^ and IKKβ^ΔFib^ littermates were infused with 1000 ng/kg/min of Ang II for 4 weeks to induce hypertensive cardiac remodeling by using osmotic minipumps (Supplemental Figure 3A) as previously described (41, 42). The infusion of Ang II did not affect the body weight or mortality rate of the mice in all groups (Supplemental Figure 3, B and C). It has been reported that Ang II infusion can cause aortic dissection or abdominal aortic aneurysm development in WT C57BL/6 mice with low incidence (43, 44). In our study, Ang II treatment led to 1 death in each genotype, likely due to aortic dissection (data not shown).

**Figure 3:**
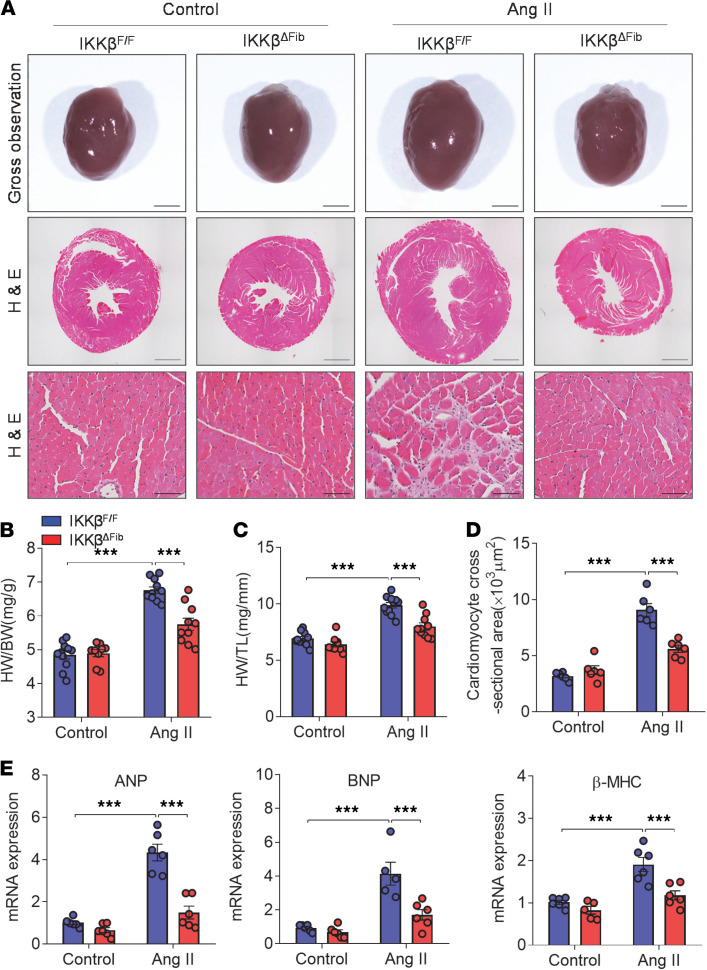
Deficiency of fibroblast IKK-β prevents angiotensin II–induced cardiac hypertrophy. Eight-week-old male IKKβ^fl/fl^ and IKKβ^ΔFib^ mice were i.p. injected with 2 mg tamoxifen per day for 5 days. At the age of 10 weeks, those mice were infused with 1000 ng/kg/min of angiotensin II (Ang II) or vehicle control for 4 weeks. (**A**) Representative photomicrographs and H&E-stained transverse sections of the hearts (scale bar for top and middle: 2000 μm; scale bar for bottom: 50 μm). (**B** and **C**) Quantitative analysis of the ratios of heart weight to body weight (**B**) or to tibial length (**C**) (*n =* 9–11; 2-way ANOVA; ***P < 0.001). (**D**) Quantification of cardiomyocyte cross-sectional area (*n =* 6; 2-way ANOVA; ***P < 0.001). (**E**) qPCR analysis of the mRNA levels of hypertrophic genes, atrial natriuretic peptide (ANP), brain natriuretic peptide (BNP), and myosin heavy chain β (β-MHC), in the hearts of IKKβ^fl/fl^ and IKKβ^ΔFib^ mice (*n =* 5–6; 2-way ANOVA; ***P < 0.001).

Although apparently phenotypically normal, IKKβ^fl/fl^ but not IKKβ^ΔFib^ mice had enlarged hearts with significantly increased heart weights and sizes after 4 weeks of Ang II infusion (Figure 3, A–C). Further analysis showed that the extent of cellular sizes as evaluated by cardiomyocyte cross-sectional area were significantly increased by Ang II infusion in IKKβ^fl/fl^ but not IKKβ^ΔFib^ mice (Figure 3D). Consistently, Ang II also induced the expression of heart hypertrophic markers, including atrial natriuretic peptide, brain natriuretic peptide, and myosin heavy chain β in IKKβ^fl/fl^ mice, and deficiency of fibroblast IKK-β blocked Ang II–induced gene expression (Figure 3E).

**Figure 4:**
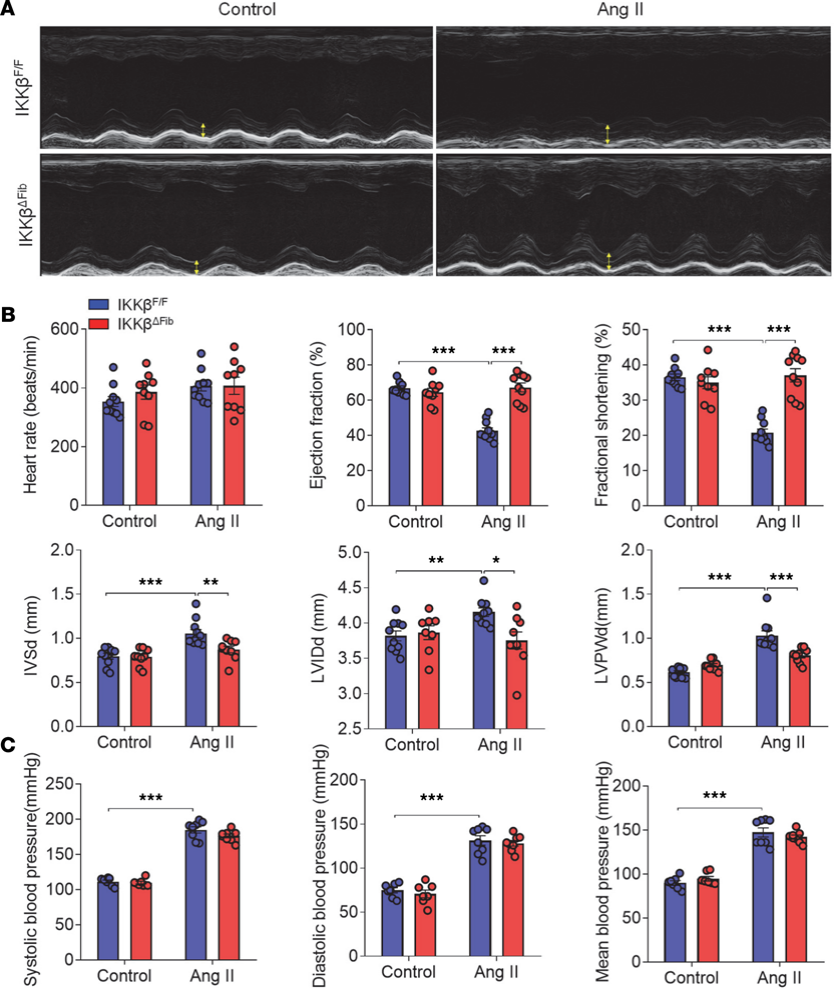
Ablation of IKK-β in fibroblasts ameliorates angiotensin II–induced cardiac dysfunction. Eight-week-old male IKKβ^fl/fl^ and IKKβ^ΔFib^ mice were i.p. injected with 2 mg tamoxifen per day for 5 days. At the age of 10 weeks, those mice were infused with 1000 ng/kg/min of angiotensin II (Ang II) or vehicle control for 4 weeks. (**A**) Representative images of M-mode echocardiography of left ventricles (LVs). (**B**) Echocardiographic analysis of heart rate (HR), ejection fraction (EF), fractional shortening (FS), interventricular septum diameter at end diastole (IVSd), LV internal dimension at end-diastole (LVIDd), and LV posterior wall at end-diastole (LVPWd) (*n =* 8–12; 2-way ANOVA; *P < 0.05, **P < 0.01, ***P < 0.001). (**C**) Measurements of systolic, diastolic, and mean blood pressure (*n =* 7–8; 2-way ANOVA; ***P < 0.001).

Hypertrophic remodeling in the heart is often associated with increased cardiac dysfunction. Under the control condition with saline infusion, IKKβ^fl/fl^ and IKKβ^ΔFib^ mice had comparable cardiac function parameters, indicating that deficiency of fibroblast IKK-β did not affect cardiac function without Ang II treatment (Figure 4, A and B and Supplemental Table 1). After 4 weeks of Ang II infusion, cardiac function had indeed deteriorated in IKKβ^fl/fl^ mice, with decreased ejection fraction and LV fractional shortening as compared with mice infused with the saline control. LV posterior wall dimension (LVPWd), LV end systolic diameter (LVSd), and LV internal dimension in diastole (LVIDd) were increased in those mice (Figure 4, A and B and Supplemental Table 1). By contrast, Ang II–induced cardiac contractile dysfunction and increased wall thickness were markedly improved in IKKβ^ΔFib^ mice (Figure 4, A and B and Supplemental Table 1).

Hemodynamic parameters were also measured, and the results showed that systolic and diastolic blood pressure and heartbeat rates were comparable between the genotypes under the control condition. Ang II treatment led to increased blood pressure and heartbeat rates in both genotypes, but deficiency of fibroblast IKK-β did not affect Ang II–elevated blood pressure and heartbeat rate (Figure 4C and Supplemental Table 1), indicating that IKK-β deficiency in cardiac fibroblasts ameliorated Ang II–induced cardiac dysfunction independent of the changes in blood pressure or heartbeat. Collectively, these results suggest that cardiac fibroblast IKK-β contributes to Ang II–induced cardiac dysfunction and hypertrophic remodeling.

### Ang II–induced cardiac fibrosis is attenuated in fibroblast-specific IKK-β–deficient mice.

**Figure 5:**
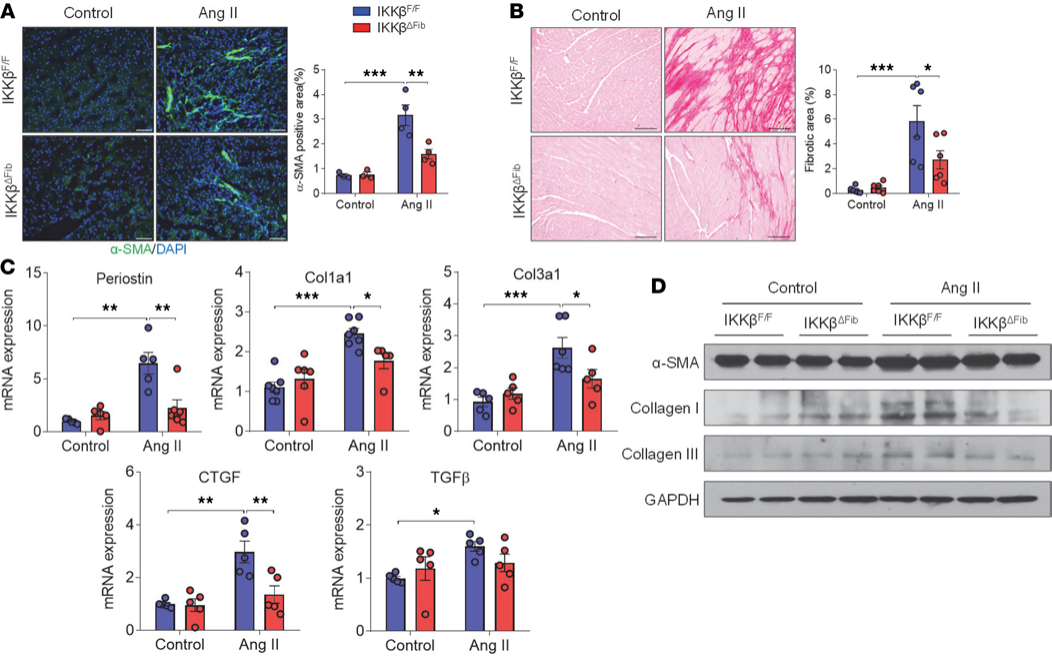
Angiotensin II–induced myocardial fibrosis was attenuated in fibroblast-specific IKK-β–deficient mice. Eight-week-old male IKKβ^fl/fl^ and IKKβ^ΔFib^ mice were i.p. injected with 2 mg tamoxifen per day for 5 days. At the age of 10 weeks, those mice were infused with 1000 ng/kg/min of angiotensin II (Ang II) or vehicle control for 4 weeks. (**A**) Representative images of immunofluorescence staining (left) and the quantification of α-SMA in the hearts of IKKβ^fl/fl^ and IKKβ^ΔFib^ mice (*n =* 3–4; 2-way ANOVA; **P < 0.01 and ***P < 0.001; scale bar: 50 μm). (**B**) Representative images of heart sections stained with Picrosirius red (left) and the quantification of interstitial fibrosis area (right) (*n =* 6, 2-way ANOVA; *P < 0.05 and ***P < 0.001; scale bar: 100 μm). (**C**) qPCR analysis of the mRNA levels of fibrotic genes in the hearts of IKKβ^fl/fl^ and IKKβ^ΔFib^ mice (*n =* 5, 2-way ANOVA; *P < 0.05, **P < 0.01, and ***P < 0.001). (**D**) Western blot analysis of α-SMA, collagen I, and collagen III proteins in the hearts of IKKβ^fl/fl^ and IKKβ^ΔFib^ mice.

We next determined the impact of fibroblast IKK-β deletion on myofibroblast differentiation and fibrosis in vivo. Immunostaining results showed that Ang II infusion increased the protein levels of myofibroblast marker α-SMA as well as extracellular matrix content in LVs of male IKKβ^fl/fl^ mice, but deficiency of fibroblast IKK-β suppressed Ang II–induced changes in male IKKβ^ΔFib^ mice (Figure 5, A and B). Consistently, mRNA levels of activated fibroblast marker periostin and several key fibrotic genes, including Col1a1, Col3a1, and connective tissue growth factor (CTGF), were upregulated by Ang II infusion in the LVs of IKKβ^fl/fl^ mice, and the induction was attenuated in IKKβ^ΔFib^ mice (Figure 5C). In addition, mRNA levels of TGF-β were also significantly upregulated by Ang II infusion in IKKβ^fl/fl^ mice (Figure 5C). Deficiency of fibroblast IKK-β led to reduced TGF-β expression in IKKβ^ΔFib^ mice, but the reduction did not reach significance because of individual variations (Figure 5C). Immunoblotting results then confirmed that Ang II–induced protein levels of α-SMA, collagen I, and collagen III in IKKβ^fl/fl^ mice were blocked in IKKβ^ΔFib^ mice (Figure 5D). Taken together, these results suggest that ablation of IKK-β in fibroblasts reduced Ang II–induced cardiac fibrotic responses in vivo.

### Ablation of fibroblast IKK-β decreases Ang II–induced cardiac fibroblast proliferation.

**Figure 6:**
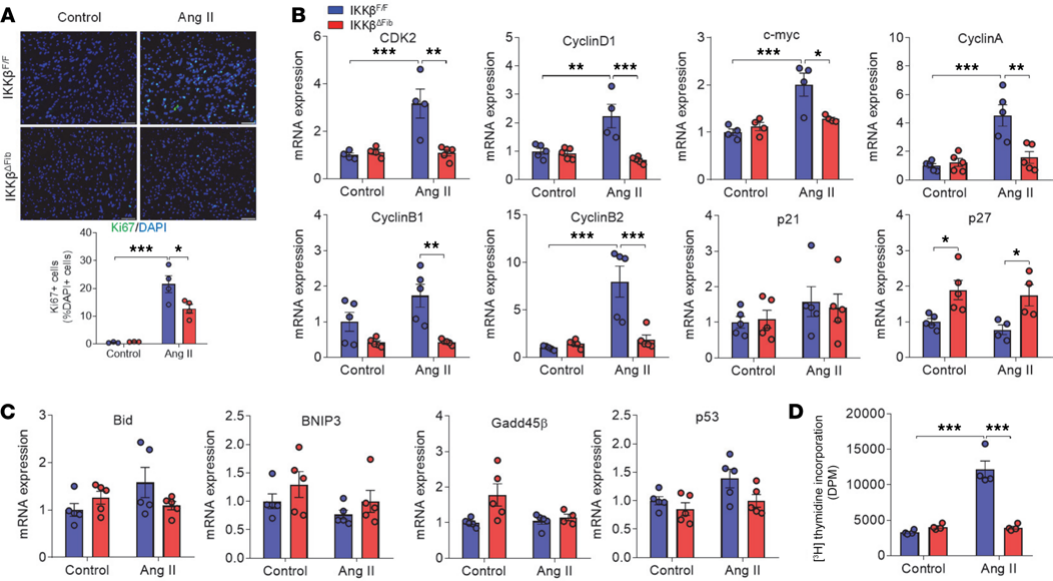
Deficiency of IKK-β decreases angiotensin II–induced cardiac fibroblast proliferation. Eight-week-old male IKKβ^fl/fl^ and IKKβ^ΔFib^ mice were i.p. injected with 2 mg tamoxifen per day for 5 days. At the age of 10 weeks, those mice were infused with 1000 ng/kg/min of angiotensin II (Ang II) or vehicle control for 1 week. (**A**) Representative images of immunofluorescence staining (top) and the quantification (bottom, normalized to the number of DAPI-stained cells) of Ki-67–positive cells in the hearts of IKKβ^fl/fl^ and IKKβ^ΔFib^ mice (*n =* 3–4, 2-way ANOVA; *P < 0.05, ***P < 0.001; scale bar: 50 μm). (**B** and **C**) qPCR analysis of the proliferation-related genes (**B**) and apoptotic genes (**C**) in the hearts of IKKβ^fl/fl^ and IKKβ^ΔFib^ mice (*n* = 4*–*5; 2-way ANOVA; *P < 0.05, **P < 0.01, ***P < 0.001). (**D**) Growth-arrested fibroblasts isolated from the hearts of IKKβ^fl/fl^ and IKKβ^ΔFib^ mice were stimulated with 10^–6^ M of Ang II or vehicle control for 24 hours, and the amount of [^3^H] thymidine incorporated into DNA of the cells was measured (*n =* 4; 2-way ANOVA; ***P < 0.001).

The proliferation of cardiac fibroblasts is also a key pathophysiological process in cardiac fibrosis (45), and enhanced proliferation and migration of cardiac fibroblasts were observed at an early stage of cardiac injury (46). Previous studies suggested that Ang II promoted fibroblast differentiation, proliferation, and inflammation at day 7 after infusion in WT mice (40, 47). To determine whether IKK-β mediates Ang II–induced cardiac fibroblast proliferation, Ki67 expression in the LVs was determined by immunofluorescence staining. As shown in Figure 6A, the number of Ki67-positive cells was significantly increased in male IKKβ^fl/fl^ mice even after 1 week of Ang II infusion, and IKK-β deletion decreased the number of proliferating cells. In adult mammals under homeostatic conditions, cardiomyocytes proliferate at an extremely low rate (48). Thus, the proliferating cells stained by Ki67 are mostly likely the cardiac fibroblasts. Ki67 costaining with a fibroblast marker (ER-TR7; ref. 49) or cardiomyocyte marker (Myh7; ref. 50) was then performed, and the results confirmed that Ki67-positive proliferating cells were indeed cardiac fibroblasts but not cardiomyocytes (Supplemental Figure 4). The mRNA levels of a list of proliferation-related genes (e.g., CDK2, cyclin D1, c-myc, cyclin A, cyclin B1, cyclinB2, p21, and P27) and apoptotic genes (e.g., Bid, BNIP3, GADD45β, and p53) in the heart were next measured. Several key genes promoting progression of the cell cycle, including CDK2, cyclin D1, c-myc, cyclin A, cyclin B1, and cyclinB2, were upregulated by Ang II infusion in IKKβ^fl/fl^ mice after Ang II infusion, which were attenuated in IKKβ^ΔFib^ mice (Figure 6B). Although IKK-β/NF-κB signaling has been known to suppress cell apoptosis, Ang II infusion and fibroblast IKK-β deficiency did not affect the mRNA levels of several genes associated with apoptosis pathways, including Bid, BNIP3, Gadd45β, and p53 (Figure 6C). We then performed TUNEL staining to check the apoptotic cells in the heart after Ang II infusion. We found that there was almost no sign of apoptotic cells in the hearts of male IKKβ^fl/fl^ and IKKβ^ΔFib^ mice after 1 week of Ang II infusion (Supplemental Figure 5A), which is consistent with a previous study demonstrating that cardiac fibroblast proliferation peaks at 4 to 7 days after pressure overload induction without signs of cardiomyocyte apoptosis (51). After 4 weeks of Ang II infusion, there were only a few apoptotic cells in the hearts of IKKβ^fl/fl^ and IKKβ^ΔFib^ mice, but deficiency of IKK-β did not lead to increased apoptosis in the heart (Supplemental Figure 5B). These results suggest that deficiency of fibroblast IKK-β inhibited the proliferation of cardiac fibroblasts without inducing apoptosis in Ang II–treated mice.

We next investigated whether IKK-β can also regulate fibroblast proliferation using cultured cardiac fibroblasts isolated from IKKβ^fl/fl^ and IKKβ^ΔFib^ mice. Consistent with in vivo results, Ang II treatment stimulated the expression of proliferation-related genes in control fibroblasts but not in IKK-β–deficient fibroblasts (Supplemental Figure 6). [^3^H]-thymidine that can be incorporated into cardiac fibroblast DNA as an index was then used to measure cell proliferation. Consistent with gene expression results, loss of IKK-β inhibited Ang II–induced DNA synthesis in cardiac fibroblasts (Figure 6D). Taken together, these data indicate that deficiency of fibroblast IKK-β inhibited cardiac fibroblast proliferation, which may contribute to improved cardiac remodeling in IKK-β–deficient mice.

### Fibroblast-specific IKK-β–deficient mice are protected from Ang II–induced cardiac inflammation.

Activated fibroblasts can also stimulate cardiac inflammation, and we next investigated the role of fibroblast IKK-β in mediating ventricular inflammation in response to Ang II infusion. We found that 4 weeks of infusion with Ang II induced the cardiac expression of proinflammatory cytokine IL-6 and adhesion molecules ICAM-1 and VCAM-1 in control male IKKβ^fl/fl^ but not IKKβ^ΔFib^ mice (Supplemental Figure 7A). However, the expression levels of other key proinflammatory genes, MCP-1 and TNF-α, and the macrophage marker F4/80 were not affected by 4 weeks of Ang II infusion (Supplemental Figure 7A). Immunostaining with antibodies against another macrophage marker, CD68, only detected a few CD68-positive cells in the hearts of IKKβ^fl/fl^ mice (Supplemental Figure 7B).

**Figure 7:**
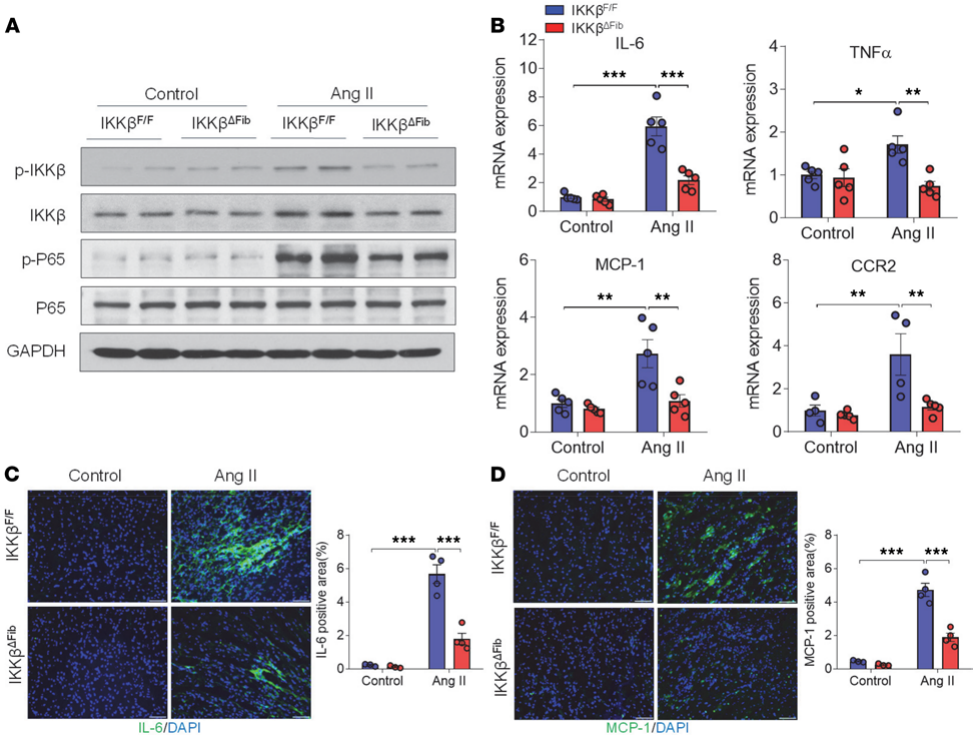
Angiotensin II–induced cardiac inflammation was inhibited in IKK-β–deficient mice. Eight-week-old male IKKβ^fl/fl^ and IKKβ^ΔFib^ mice were i.p. injected with 2 mg tamoxifen per day for 5 days. At the age of 10 weeks, those mice were infused with 1000 ng/kg/min of angiotensin II (Ang II) or vehicle control for 1 week. (**A**) Western blot analysis of the protein levels of total and phosphorylated (p) IKK-β and NF-κB subunit p65 in the hearts of IKKβ^fl/fl^ and IKKβ^ΔFib^ mice. (**B**) qPCR analysis of the mRNA levels of inflammatory cytokines and chemokines in the hearts of IKKβ^fl/fl^ and IKKβ^ΔFib^ mice (*n* = 4–5; 2-way ANOVA; *P < 0.05, **P < 0.01, ***P < 0.001). (**C** and **D**) Representative images of immunofluorescence staining (left) and the quantifications (right) of IL-6 (**C**) and MCP-1 (**D**) in the hearts of IKKβ^fl/fl^ and IKKβ^ΔFib^ mice. The nuclei were visualized with DAPI (blue) (*n =* 3–4; 2-way ANOVA; ***P < 0.001; scale bar: 50 μm).

To determine whether Ang II activates fibroblast IKK-β/NF-κB signaling to induce inflammatory responses at a relatively early stage, Ang II was infused into control and fibroblast-specific IKK-β–deficient mice for 1 week. Short-term Ang II infusion was able to increase the phosphorylated IKK-β and p65 protein levels in the hearts of IKKβ^fl/fl^ mice, but the induction was reduced in IKKβ^ΔFib^ mice (Figure 7A). Consistent with elevated NF-κB activation, Ang II infusion significantly increased the expression levels of several key inflammatory genes, including IL-6, MCP-1, and TNF-α, in the hearts of IKKβ^fl/fl^ mice, and deficiency of fibroblast IKK-β attenuated these gene expression levels in IKKβ^ΔFib^ mice (Figure 7B). Further, immunostaining results confirmed the robustly increased cardiac MCP-1 and IL-6 protein levels in IKKβ^fl/fl^ but not in IKKβ^ΔFib^ mice (Figure 7, C and D).

### Fibroblast IKK-β contributes to Ang II–induced cardiac macrophage infiltration.

**Figure 8:**
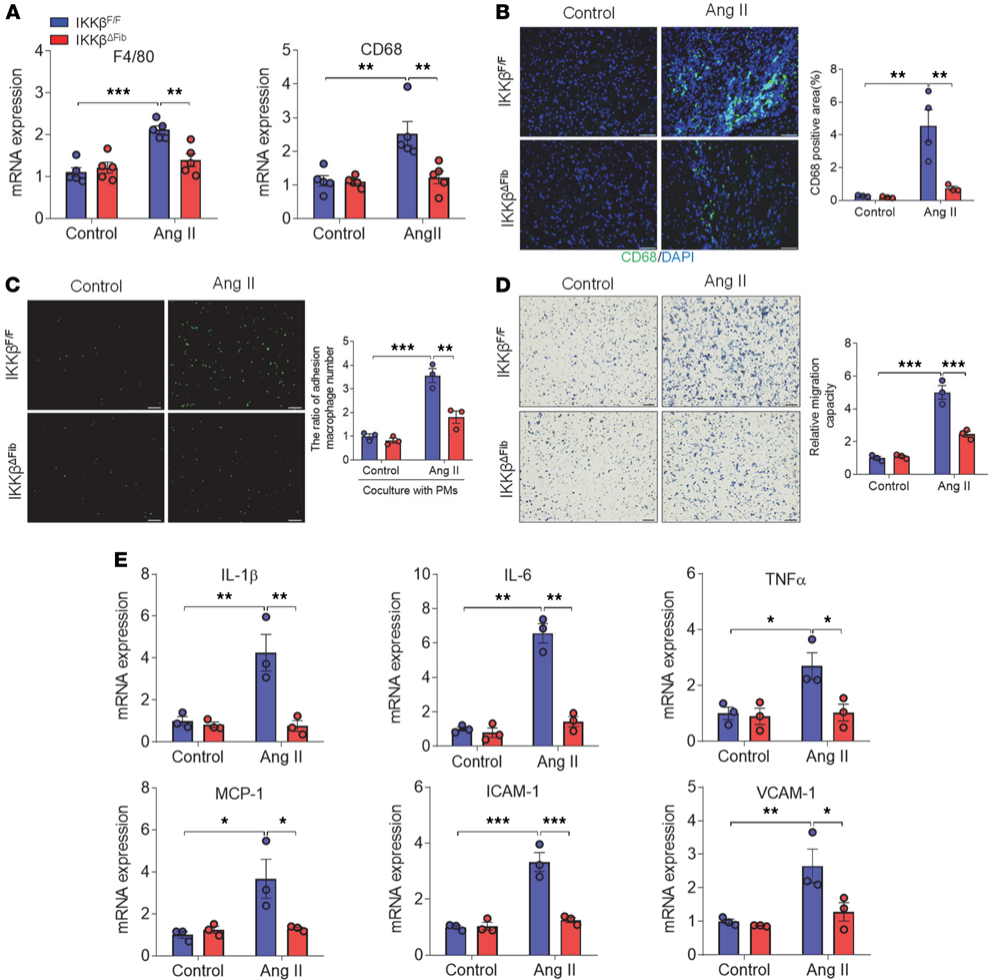
Deficiency of fibroblast IKK-β reduces angiotensin II–elicited cardiac macrophage infiltration. (**A** and **B**) Eight-week-old male IKKβ^fl/fl^ and IKKβ^ΔFib^ mice were i.p. injected with 2 mg tamoxifen per day for 5 days. At the age of 10 weeks, those mice were infused with 1000 ng/kg/min of angiotensin II (Ang II) or vehicle control for 1 week. qPCR analysis of the mRNA levels of macrophage markers in the hearts of IKKβ^fl/fl^ and IKKβ^ΔFib^ mice (*n =* 5, 2-way ANOVA; **P < 0.01 and ***P < 0.001) (**A**). Representative images of immunofluorescence staining (left) and the quantification (right) of CD68 in the hearts of IKKβ^fl/fl^ and IKKβ^ΔFib^ mice (*n =* 3–4, 2-way ANOVA; **P < 0.01; scale bar: 50 μm). The nuclei were visualized with DAPI (blue) (**B**). (**C**) Cardiac fibroblasts (CFs) were isolated from IKKβ^fl/fl^ or IKKβ^ΔFib^ mice and pretreated with 10^–6^ M of Ang II for 24 hours. CFs were cocultured with calcein acetoxymethyl–labeled peritoneal macrophages (PMs) from IKKβ^fl/fl^ mice for 2 hours. Adhered PMs were counted under a fluorescence microscope. Representative images (left) and the quantification (right) of adhered PMs were presented (*n =* 3; 2-way ANOVA; **P < 0.01, ***P < 0.001; scale bar: 200 μm). (**D**) PMs of IKKβ^fl/fl^ mice were seeded on the Matrigel-coated Transwell filters for 24 hours. The lower chambers were filled with the conditioned medium from cultured control or IKK-β–deficient CFs treated with vehicle control or 10^–6^ M of Ang II. PMs that infiltrated and migrated to the underside of Transwells were stained with hematoxylin and counted under the microscope. Representative images (left) and the quantification (right) of migrated PMs (*n =* 3, 2-way ANOVA; ***P < 0.001; scale bar: 100 μm). (**E**) PMs isolated from IKKβ^fl/fl^ were treated with conditioned medium from cultured control or IKK-β–deficient CFs treated with vehicle control or 10^–6^ M of Ang II. qPCR analysis was performed to measure the mRNA levels of inflammatory cytokines and adhesion molecules (*n =* 3, 2-way ANOVA; *P < 0.05, **P < 0.01, and ***P < 0.001).

In addition to resident cardiac cells, nonresident immune cells, such as macrophages, also contribute to cardiac inflammatory responses after overload (8, 52, 53). Next, cardiac macrophage infiltration in male IKKβ^fl/fl^ and IKKβ^ΔFib^ mice was investigated, and the results indicated that Ang II infusion significantly increased the expression levels of macrophage markers F4/80 and CD68 in the hearts of IKKβ^fl/fl^ but not IKKβ^ΔFib^ mice (Figure 8A). Consistently, immunofluorescence staining showed a substantially increased CD68-positive area in the hearts of IKKβ^fl/fl^ mice after Ang II infusion, but deficiency of IKK-β blocked most Ang II–stimulated CD68 induction in IKKβ^ΔFib^ mice (Figure 8B).

To determine how fibroblast IKK-β deficiency reduced macrophage infiltration into cardiac tissues, in vitro macrophage adhesion and migration assays were performed. For adhesion assays, peritoneal macrophages isolated from control mice were labeled with calcein acetoxymethyl and then incubated with monolayers of control or IKK-β–deficient cardiac fibroblasts that were treated with vehicle control or Ang II. As shown in Figure 8C, Ang II–treated fibroblasts of IKKβ^fl/fl^ mice recruited more macrophages as compared with fibroblasts of IKKβ^ΔFib^ mice. Next, Transwell migration assays were also performed by using conditioned medium collected from control or Ang II–treated cardiac fibroblasts of IKKβ^fl/fl^ or IKKβ^ΔFib^ mice. Conditioned medium collected from Ang II–treated cardiac fibroblasts of IKKβ^fl/fl^ mice enhanced the macrophage migration properties, but the increased migration was suppressed when using conditioned medium collected from Ang II–treated IKK-β–deficient fibroblasts (Figure 8D).

Lastly, qPCR analysis demonstrated that conditioned medium from Ang II–treated control fibroblasts also significantly upregulated mRNA levels of proinflammatory genes and key adhesion molecules, including IL-6, MCP-1, TNF-α, ICAM-1, and VCAM-1, in macrophages as compared with control medium-treated cells. However, conditioned medium from Ang II–treated IKK-β–deficient fibroblasts abolished the observed upregulation (Figure 8E). These results suggest that Ang II–mediated fibroblast IKK-β activation promoted macrophage adhesion and migration properties, leading to increased macrophage infiltration into cardiac tissue.

## Discussion

**Figure 9:**
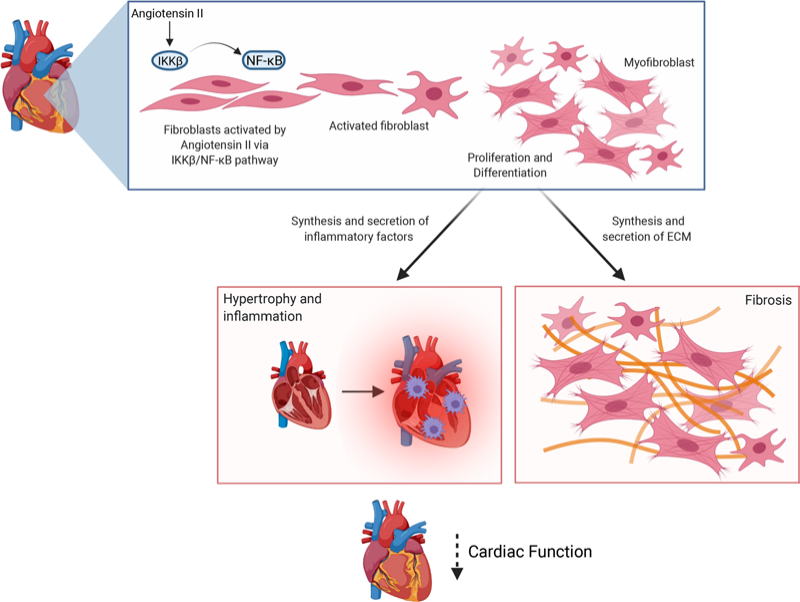
Schematic representation of the role of fibroblast IKK-β in mediating angiotensin II–elicited adverse cardiac remodeling and cardiac dysfunction. Activation of IKK-β by angiotensin II (Ang II) stimulates cardiac fibroblast proinflammatory and profibrogenic responses, leading to increased cardiac fibrosis, hypertrophy, and macrophage infiltration. As a consequence, fibroblast IKK-β signaling contributes significantly to Ang II–induced adverse cardiac remodeling and cardiac dysfunction.

Cardiac fibroblasts have been indicated as sentinel cells that can interact with local cardiomyocytes and inflammatory cells to regulate cardiac remodeling and functions (29, 54). As a central coordinator of inflammation and immune responses through activation of NF-κB, the role of IKK-β in regulating cardiac fibroblast function and cardiac remodeling has not been well studied. In the present study, we generated inducible fibroblast-specific IKK-β–knockout mice and demonstrated that fibroblast IKK-β contributed significantly to the Ang II–induced cardiac inflammation and adverse remodeling in male mice. Deficiency of IKK-β inhibited Ang II–stimulated fibroblast proliferation, differentiation, and fibrogenesis. In addition to regulating cardiac fibrosis, fibroblasts can secrete a wide array of cytokines and chemokines that modulate macrophage activity to amplify inflammatory responses (31–33). Indeed, activation of IKK-β by Ang II treatment also led to increased macrophage infiltration and elevated cardiac inflammation, which may coordinately promote adverse cardiac remodeling and dysfunction in Ang II–treated mice. By contrast, deficiency of fibroblast IKK-β protected mice from Ang II–induced cardiac inflammation, fibrosis, and dysfunction. Our results suggest that fibroblast IKK-β plays an important role in the pathogenesis of adverse cardiac remodeling and dysfunction (Figure 9).

Although the functions of IKK-β in vascular diseases such as atherosclerosis have been well studied, the role of IKK-β in the regulation of cardiac remodeling and functions remain elusive. Previous studies targeting cardiomyocyte IKK-β have generated inconsistent results (26, 28, 55). In addition to cardiomyocytes, cardiac fibroblasts also play a key role in cardiac remodeling and fibrosis (56). Under normal physiological conditions, cardiac fibroblasts provide the mechanical scaffold for cardiac myocytes and coordinate cardiac pump function by regulating cardiac extracellular matrix homeostasis (56, 57). In response to other conditions, such as inflammation, hypertension, and myocardial injury, fibroblasts can transdifferentiate into pathological activated myofibroblasts that secrete excessive amounts of matrix molecules, leading to accumulation of cardiac collagen and eventually cardiac dysfunction (29, 58). Inhibition of IKK-β has been previously shown to suppress TGF-β–induced transition of fibroblasts into myofibroblasts and the synthesis of extracellular matrix in human dermal and lung fibroblasts (34). In addition, selective deletion of IKK-β in mouse airway epithelium also led to less peribranchial fibrosis (59). Consistent with these studies, our study showed that Cre-mediated IKK-β deletion or pharmacological inhibition of IKK-β inhibited Ang II–induced fibroblast differentiation into myofibroblasts and collagen synthesis in vitro.

Using transgenic mice expressing tamoxifen-inducible Cre recombinase driven by the Col1a2 promoter, we generated fibroblast-specific IKK-β–knockout mice and confirmed that deficiency of IKK-β reduced the Ang II–induced fibrotic gene expression and cardiac fibrosis. However, similar to many Cre transgenic models, Co1a2 is not expressed only by the fibroblasts in the heart. As one of the most populous nonmyocyte cell types in the heart, cardiac fibroblasts have been well-established to play an important role in mediating cardiac fibrosis and remodeling (7, 29). It is unlikely that fibroblasts at other tissues contributed to the observed phenotypes in our models. A previous lineage-tracing study identified the resident cardiac fibroblasts as the main source for activated myofibroblasts in the injured heart (60). In addition, it has been demonstrated that more than 96% of Col1a2-positive cells in the heart were fibroblasts but not other cell types (61). Col1a2-positive cells have also been shown to be colocalized with other fibroblast markers but not with cardiomyocyte, endothelial, or smooth muscle cell markers (40). These studies strongly suggest that Col1a2-positive cells in the heart are cardiac fibroblasts that contribute to the observed phenotypes in our study.

Ang II–induced high blood pressure plays an important role in the pathogenesis of cardiac remodeling by increasing pressure overload, and sex differences in blood pressure levels have been well recognized (62). The blood pressure differences between females and males have been attributed, at least partially, to sex hormones (62). To avoid the potential sex hormone–involved effects, our current study selected male mice as a model to study the role of IKK-β signaling in mediating Ang II–induced cardiac remodeling, which is also a limitation of this study. It would be interesting to study whether fibroblast IKK-β may have sex-specific effects on cardiac remodeling in the future.

In addition to myofibroblast differentiation, proliferation of activated cardiac fibroblasts also plays an important role in increasing proinflammatory and profibrotic effects induced by Ang II (29). IKK-β/NF-κB signaling is also one of the key pathways that regulate cell proliferation and apoptosis (63). Consistently, our studies demonstrated that deletion of IKK-β markedly decreased Ang II–induced cell proliferation marker Ki67 and proliferation-related gene expression without affecting cell apoptotic markers. TUNEL staining also demonstrated that deficiency of IKK-β did not lead to increased cell apoptosis in the heart under our experimental condition. Further, an in vitro cell proliferation assay confirmed that loss of IKK-β inhibited Ang II–induced DNA synthesis in cardiac fibroblasts. These results suggest that inhibition of cardiac fibroblast proliferation may also contribute to ameliorated cardiac remodeling and reduced cardiac hypertrophy in Ang II–treated IKKβ^ΔFib^ mice.

In addition to regulating fibrosis, fibroblasts and their pathological counterpart myofibroblasts are versatile cells that can interact with other cell types in the heart, including cardiomyocytes and macrophages (64, 65). Cardiac fibroblasts have been shown to produce proinflammatory mediators that promote transendothelial migration of monocytes into the cardiac tissue, leading to increased monocyte recruitment and aggravated inflammation (32). Several recent studies have demonstrated that monocytes and macrophages are critical early mediators of Ang II–induced vascular dysfunction, arterial hypertension, and cardiac fibrosis in mice (52, 53, 66). Ang II–induced monocyte infiltration and elevated proinflammatory genes in the heart can occur as early as 3 hours to 1 day after Ang II infusion. For example, a recent study showed that macrophage infiltration happened at day 1 of Ang II infusion and remained elevated for 14 days after Ang II infusion, with the peak observed at day 7 (67). Consistent with those studies, we also observed robust recruitment of macrophages to the heart after 1 week of Ang II infusion, and deficiency of fibroblast IKK-β substantially reduced cardiac macrophage infiltration and inflammation. Chemokine gradients are required to recruit macrophages to cardiac tissue, and one of the important sources of these chemoattractive substances is activated fibroblasts (53). Indeed, we found that Ang II–treated control fibroblasts increased macrophage adhesion and migration properties, but deficiency of IKK-β abolished the impact of fibroblasts on macrophage adhesion and migrations. Further, the conditioned medium from Ang II–treated control fibroblasts but not IKK-β–deficient fibroblasts led to increased expression of proinflammatory cytokines and chemokines in macrophages. Therefore, activation of fibroblast IKK-β signaling led to increased monocytes infiltrating into cardiac tissue, and those re-infiltrated monocytes may further enhance the vicious circle of cardiac inflammation. Our current study showed the impact of fibroblast IKK-β deficiency on Ang II–induced macrophage infiltration into the heart and on inflammatory responses, but the specific contribution of different types of macrophages (e.g., CCR2^+^ vs. CCR2^–^) toward Ang II–induced cardiomyopathy will need further investigation. It would also be interesting to study whether depletion of macrophages can ameliorate Ang II–induced cardiac fibrosis and dysfunction in the future. Results from our study will hopefully stimulate future investigation on how IKK-β signaling in different cell types (e.g., cardiomyocytes, fibroblasts) regulates different types of macrophages infiltrating into the heart and their contribution to cardiac fibrosis and dysfunction in hypertensive cardiac remodeling.

In summary, we generated inducible fibroblast-specific IKK-β–knockout mice to investigate the contribution of fibroblast IKK-β signaling toward Ang II–induced adverse cardiac remodeling and cardiac dysfunction. We found that deficiency of fibroblast IKK-β protected male mice from Ang II–induced cardiac inflammation, fibrosis, and dysfunction. There results demonstrated a pivotal role of fibroblast IKK-β in regulating hypertension-related cardiac remodeling, and targeting fibroblast IKK-β may represent a therapeutic approach against adverse cardiac remodeling and dysfunction. Findings from previous studies and the current one also suggest that functions of IKK-β signaling in cardiovascular disease are complex, and further investigations are required to dissect the cell type–specific role of IKK-β in regulating cardiac remodeling and function.

## Methods

### Generation of inducible fibroblast-specific IKK-β–deficient mice.

The Col1a2CreER^T^ mice on C57BL/6J background were obtained from The Jackson Laboratory (stock 029567). The transgenic mice expressed tamoxifen-inducible Cre recombinase driven by the mouse Col1a2 promoter (40, 68). Mice carrying loxP-ﬂanked IKK-β alleles (IKKβ^fl/fl^) on C57BL/6J background have been described before (11, 16, 19). IKKβ^fl/fl^ mice were bred with Col1a2CreER^T^ mice to generate inducible fibroblast-specific IKKβ^fl/fl^-deficient (IKKβ^ΔFib^) mice. Tamoxifen (2 mg) (Sigma-Aldrich, T5648) dissolved in 1 mL corn oil was i.p. injected for 5 consecutive days to induce Cre-mediated recombination. One week after the completion of tamoxifen dosing (to allow the clearance of tamoxifen), the mice were used for further experiments. All mice used in this study had IKKβ^fl/fl^ background, and IKKβ^ΔFib^ mice carried heterozygous knockin for tamoxifen-inducible Cre recombinase. All experimental mice used in this study were male littermates, partially due to the known crosstalk between NF-κB and estrogen signaling (69, 70). However, the authors are aware of the fact that studying a single sex has limitations since sex differences have been widely reported in mouse studies.

### Ang II infusion and blood pressure measurement.

Eight-week-old male IKKβ^fl/fl^ and IKKβ^ΔFib^ littermates were i.p. injected with 2 mg tamoxifen per day for 5 days. At the age of 10 weeks, those mice were implanted with mini-osmotic pumps (ALZET model 2004; DURECT Corporation) containing Ang II (1000 ng/kg/min; Bachem Americas Inc., 4006473) or vehicle control (normal saline) for 1 or 4 weeks as previously described (40, 53, 67). Brieﬂy, a 1.0 cm vertical midscapular skin incision was made in mice anesthetized with isoflurane, followed by creation of a 3.5 cm deep pocket, where a mini-osmotic pump was inserted. Skin closure was performed using 4-0 silk sutures, and mice were allowed to recover on a heating plate at 37°C. Blood pressure was measured by noninvasive tail-cuff method (Kent Coda 8; Kent Scientific Corporation) on a preheated 37°C plate to dilate the tail artery, as previously described (71). The average of no less than 5 successive measurements for each mouse was considered as the individual blood pressure.

### Echocardiography.

Transthoracic echocardiography was performed with a high-resolution microimaging system equipped with a 30 MHz transducer (Vevo3100; Visual Sonics Inc.; ref. 72). Briefly, after isoflurane inhalation, cardiac echocardiography of saline- or Ang II–infused male mice was recorded on a heating plate at 37°C. M-mode cardiac images of the LV from the short-axis view were used to assess LV morphological parameters, end-systolic interventricular septum diameter, end-diastolic LVPW thickness, LV end-diastolic dimension and LV end-systolic dimension, and LV mass, as well as LV systolic parameters of ejection fraction, and fractional shortening (73).

### Western blotting.

Western blotting was performed as previously described (19). Brieﬂy, cell or tissue lysate samples were resolved on SDS-PAGE. Proteins were then transferred to nitrocellulose membrane. The membrane was blocked in PBS solution with 0.05% Tween 20 (PBST, pH 7.4) containing 5% BSA (MilliporeSigma, A9647) for 1 to 3 hours and then incubated with primary antibody in PBST containing 5% BSA at 4°C overnight. After the incubation, the membrane was washed 4 times with PBST and incubated with secondary antibody in PBST with 5% nonfat dry milk (Bio-Rad, 170-6404) for 1 hour at room temperature. After washing 3 times in PBST, the membrane was washed once in PBS and developed using Pierce ECL Western blotting substrate (Thermo Fisher Scientific, 32209) and exposed to CL-XPosure films (Thermo Fisher Scientific, 34099). Western blots were probed with the primary antibodies as follows: anti-GAPDH (Sigma-Aldrich, G9545, 1:5000), anti-actin (MilliporeSigma, A2066, 1:5000); anti–IKK-β (Cell Signaling Technology, 2678, 1:1000), anti–phosphorylated IKK-β (Ser176/180, Cell Signaling Technology, 2697, 1:1000), anti–NF-κB P65 (Cell Signaling Technology, 3034, 1:1000), anti–phosphorylated NF-κB P65 (Ser536, Cell Signaling Technology, 3033, 1:1000), anti–collagen I (Abcam, ab34710, 1:1000), anti–collagen III (Abcam, ab7778, 1:1000), anti–α-SMA (Abcam, ab5694, 1:4000), and anti–MCP-1 (Abcam, ab7202, 1:1000).

### RNA isolation and qPCR.

Total RNA was extracted from mouse tissues or cells using TRIzol reagent (Thermo Fisher Scientific, 15596026), and total RNA was converted to cDNA using the Script Reverse Transcription Supermix Kit (Invitrogen). qPCR was performed using gene-specific primers and the SYBR Green PCR kit (Bio-Rad, 170-8886) as previously described (16, 19). The sequences of primer sets used in this study are listed in Supplemental Table 2. Gene expression levels were normalized with the housekeeping gene GAPDH. All samples were run in duplicate. The relative fold change was computed by the ^ΔΔ^Ct method.

### Histopathology analysis.

The hearts of saline or Ang II–infused male mice were isolated, fixed with 10% formaldehyde in PBS at room temperature for 24 hours, embedded in paraffin, and sectioned at the thickness of 6 μm. Heart morphological histomorphometric characters were analyzed via H&E staining. Picrosirius red stain kit (Polysciences Inc.) was performed to identify collagen fibers by following the manufacturer’s instructions. H&E staining and Picrosirius red–stained interstitial fibrosis was observed with light microscopy (Nikon) at ×20 or ×40 magnification. The digital photomicrographs were quantified with Image-Pro Plus Software (Media Cybernetics). Cardiomyocyte size was assessed on H&E-stained sections. About 100 randomly chosen cardiomyocytes for each mouse sample were analyzed to measure cross-sectional cardiomyocyte area. The percentage area of myocardial interstitial fibrosis area was evaluated by Picrosirius red–stained sections. About 5 random fields within the midmyocardium in each murine sample were assayed in order to exclude large epicardial arteries/veins and any cutting/compression artifacts.

### Immunofluorescence staining.

The cryo-sections of mouse LV and cultured mouse primary cells were used for immunofluorescence staining in this study. The hearts of the mice were freshly embedded in OCT and sectioned at the thickness of 7 μm. The heart sections or cells were first fixed in 4% PFA for 15 minutes and washed with PBS for 10 minutes. Then, the samples were permeabilized with 0.1% Triton X-100 in PBS for 10 minutes. Nonspecific binding was reduced by the incubation of 10% rabbit sera diluted in PBST for 20 minutes at room temperature. The samples were then incubated with antibodies against CD68 (Bio-Rad AbD Serotec, MCA1957), rabbit MCP-1 (Abcam, ab7202), IL-6 (Bio-Rad AbD Serotec, MCA1490), α-SMA (Abcam, ab5694), collagen I (Abcam, ab34710), ER-TR7 (Novus Biologicals, NB100-64932), Myh7 (Abcam,ab50967), or Ki67 (Abcam, ab15580) at 4°C for 12 to 15 hours. The sections or cells were rinsed with PBS and incubated with fluorescein-labeled secondary antibodies (Life Technologies). The nuclei were stained by mounting the slides with DAPI medium (Vector Laboratories). TUNEL staining was performed using the In Situ Cell Death Detection Kit (Roche Applied Science) as previously described (14). Images were acquired using Nikon fluorescence microscopy.

### Isolation of cardiac fibroblasts.

Adult cardiac fibroblasts were isolated from cardiac ventricles of 10-week-old male IKKβ^fl/fl^ and IKKβ^ΔFib^ mice and their littermate control mice after tamoxifen injection. The mice were euthanized by i.p. injection of ketamine. The heart samples were excised immediately after the mice were euthanized, minced to 1 to 2 mm pieces, and digested with 200 U/mL type II collagenase (Worthington Biochemical) in HBSS at 37°C for 15 minutes by constant stirring. After undigested heart tissues were allowed to settle, the supernatant from the first digestion, containing large amounts of cell debris and blood cells, was discarded. At the end of the subsequent second digestion by type II collagenase, the supernatant, composed of mostly cardiac fibroblasts, was carefully aspirated, transferred to a fresh tube, centrifuged at 500*g* for 5 minutes, and resuspended in DMEM/F12 with 10% FBS and 1% penicillin/streptomycin (P/S, Invitrogen). This digestion was repeated 6 to 8 times in the same way until the digestion solution became clear. Cells were plated on gelatin-coated 25 cm^2^ flasks. After incubation for 2 hours at 37°C, allowing cardiac fibroblasts to attach to the bottom of the plate, unattached cells, including primary cardiomyocytes, endothelial cells, and leukocytes, were washed away. Cardiac fibroblasts were cultured in DMEM with 10% FBS and 1% P/S at 37°C in a humidified atmosphere of 5% CO_2_ and 95% air. The cardiac fibroblasts at passage 2 to 3 were used for further experiments (73). For macrophage studies, cardiac fibroblasts were serum starved and stimulated for 24 hours with 10^–6^ M Ang II. The mixture of supernatant in DMEM was collected and filtered. Peritoneal macrophages isolated from IKKβ^fl/fl^ mice were serum-starved and then cocultured for 24 hours in 1:1 mixed medium of serum-free DMEM and the conditioned medium from cardiac fibroblasts.

### Virus production and transduction.

Lentivirus particles expressing lacZ control and Cre were produced as previously described (19). Briefly, for lentivirus production and amplification, pPACKH1 HIV Lentivector Packaging Kit (System Biosciences, LV500A-1) was used according to the manufacturer’s instructions. The plasmids for lentiviral-Cre (LV-Cre, 12106) and lentiviral-LacZ (LV-lac, 12108) were purchased from Addgene. For the virus-mediated deletion, cells in 6-well plates were washed twice with PBS and treated with 2 mL of serum-free media containing 5 mg/mL of polybrene and virus. The virus-containing medium was refreshed every day. After 2 days of overnight incubation, the virus-containing medium was removed and replaced with fresh complete medium. The cells were used for further experiments the next day.

### Thymidine assay for proliferation analysis.

Proliferation assay of thymidine was performed according to the known method (74). Briefly, the cardiac fibroblasts were plated in 6-well plates at a density of 7 × 10^4^ cells/well with DMEM/F12 containing 10% serum for 24 hours and then starved in 0.5% serum for 24 hours. Cells were treated with 10^–6^ M Ang II. For all the experimental groups, cardiac fibroblasts were exposed to 1 μCi (PerkinElmer Life Sciences) per plate of [^3^H] thymidine for the last 6 hours in the 24-hour incubation period with Ang II. At the end of this period, plates were placed on ice, and cells were washed 3 times each with ice-cold PBS, then twice with ice cold 10% trichloroacetic acid, and finally with ethanol/ether (2:1). The cells were dissolved with 0.1% SDS in 0.1 N NaOH by incubating overnight at room temperature. [^3^H] thymidine was quantified in a liquid scintillation counter (Packard 2200CA, Packard Instrument Co.).

### Macrophage adhesion and migration assays.

Peritoneal macrophages were isolated as previously described (75). Peritoneal macrophages (5 × 10^6^) were resuspended with 1 mL of DMEM/F12 and labeled with fluorescent calcein acetoxymethyl (5 μM) for 30 minutes at 37°C. Labeled peritoneal macrophages were added to 24-well microplates containing a layer of 2 × 10^4^ cardiac fibroblasts in each well that had previously been untreated or treated with 100 ng/mL LPS for 24 hours or 10^–6^ M Ang II for 48 hours. After coincubation for 2 hours at 37°C, nonadherent cells were removed by washing 4 times with DMEM/F12. Finally, 200 μL of PBS was added to each well and peritoneal macrophages adhered to cardiac fibroblasts were counted under the fluorescence microscope (Nikon). Macrophage migration assays were performed using Transwells with 8.0 μm pore polycarbonate membrane inserts (Thermo Fisher Scientific, 07-200-165). Two hundred microliter Matrigel (Corning, 356327, final concentration: 300 μg/mL) was added to a 24-well Transwell insert and solidified in a 37°C incubator for 2 hours to form a thin gel layer. Peritoneal macrophages were seeded on top of the Matrigel-coated Transwell filters, and the lower chambers were filled with the conditioned medium from untreated or Ang II–pretreated fibroblasts. After 24 hours, cells were removed from the upper surface of the insert by scraping using q-tips. The membranes were fixed with 100% cold methanol (Thermo Fisher Scientific, A412-4), stained with hematoxylin (Leica, 3801575), and mounted on the slides using glycerol gelatin. Hematoxylin-stained cells were counted under the microscope (Nikon) (75).

### Statistics.

All data are presented as the mean ± SEM. Individual pairwise comparisons were analyzed by 2-sample, 2-tailed Student’s *t* test unless otherwise noted, with a *P* value less than 0.05 regarded as significant. The statistical significance of differences among more than 2 groups was assessed using 1-way ANOVA with Dunnett’s test. Two-way ANOVA was used when multiple comparisons were made, followed by a Bonferroni multiple-comparison test. Two-way ANOVA was done using SigmaPlot 13.0. The other statistics were analyzed using GraphPad Prism 7.0.

### Study approval.

All animal studies were performed in compliance with the IACUC protocols approved by the University of Kentucky and the University of California, Riverside.

## Author contributions

CZ and WL conceptualized and designed the research. WL performed most of the experiments and analyzed the data with help from ZM and RH. WL, ZM, RH, and CZ wrote the manuscript.

## Supplementary Material

Supplemental data

## Version 1 07/29/2021

In-Press Preview

## Version 2 09/22/2021

Electronic publication
