# Supplementary material for: Fibroblast-specific IKK-β deficiency ameliorates angiotensin II–induced adverse cardiac remodeling in mice
Source: JCI Insight. 2021 Sep 22;6(18):e150161. doi: 10.1172/jci.insight.150161 (PMC8492299; doi:10.1172/jci.insight.150161)
Supplement: Supplemental data [file jciinsight-6-150161-s125.pdf]

Supplemental Material for

Fibroblast-specific IKKβ deficiency ameliorates angiotensin II-induced adverse cardiac remodeling in mice

Weiwei Lu,[1] Zhaojie Meng,[2] Rebecca Hernandez,[2] and Changcheng Zhou[2]

[1]Department of Pharmacology and Nutritional Sciences, College of Medicine, University of Kentucky, Lexington, Kentucky, USA.

[2]Division of Biomedical Sciences, School of Medicine, University of California, Riverside, California, USA.

This PDF file includes:

Supplemental Figure 1-7
Supplemental Table 1-2

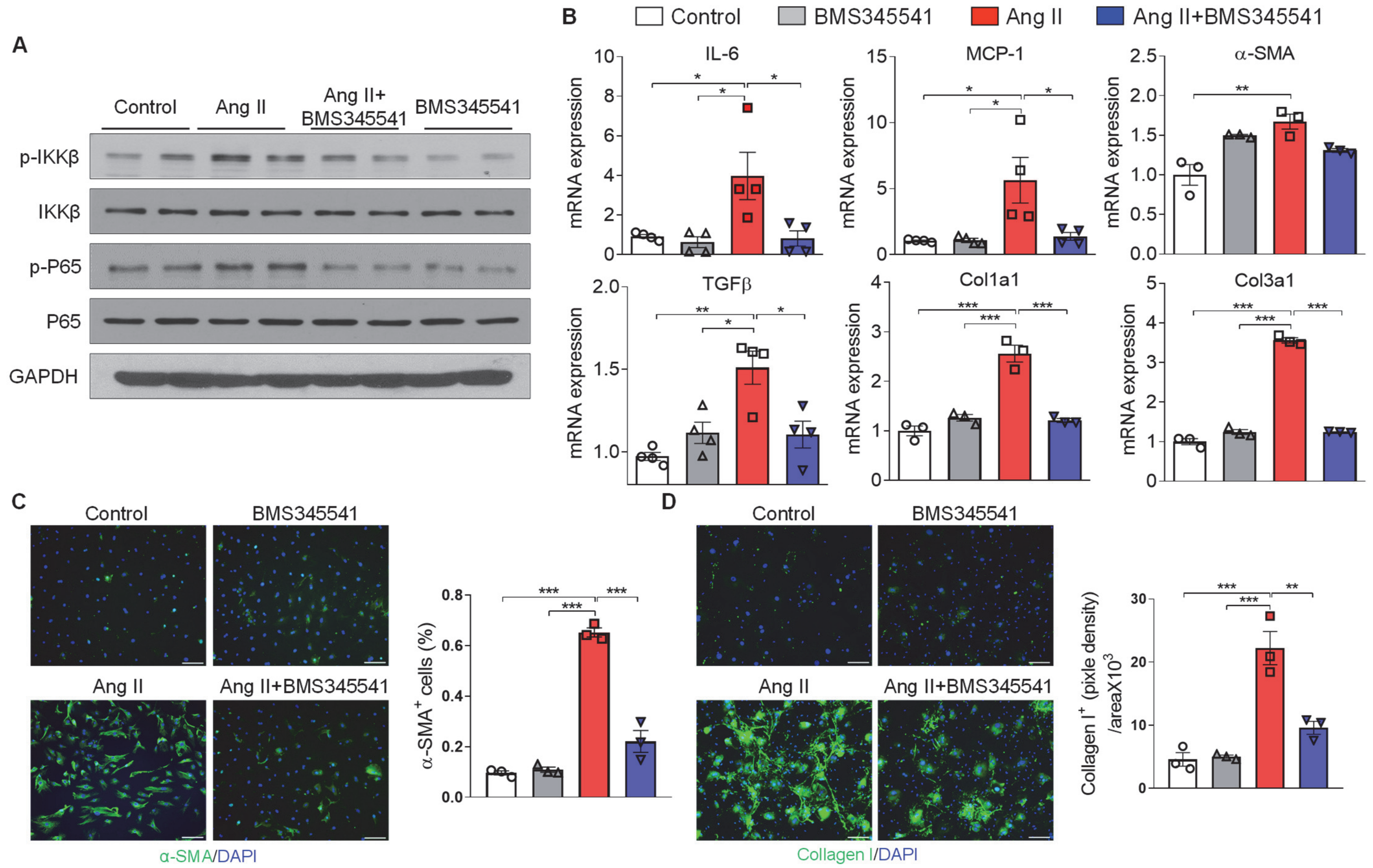

Supplemental Figure 1. Pharmacological inhibition of IKKβ signaling reduces angiotensin II-induced fibroblast proinflammatory and profibrogenic effects in vitro.

Cardiac fibroblasts (CFs) isolated from male IKKβ$^{F/F}$ mice were pretreated with 5 μM of IKKβ inhibitor BMS-345541 or vehicle control for 1 hr, and then incubated with 10^{-6} M of angiotensin (Ang) II or vehicle control for 24 hr. (A) Western blot analysis of the total and phosphorylated (p) IKKβ and NF-κB subunit p65. (B) QPCR analysis of the mRNA levels of proinflammatory cytokines and profibrotic genes (n=3-4, One-way ANOVA, *$P < 0.05$, **$P < 0.01$, and ***$P < 0.001$). (C and D) Representative immunofluorescent images (left) and quantitation (right) of α-SMA$^+$ cells (C) and collagen I (D) (n=3, One-way ANOVA, **$P < 0.01$ and ***$P < 0.001$; Scale bar, 100 μm).

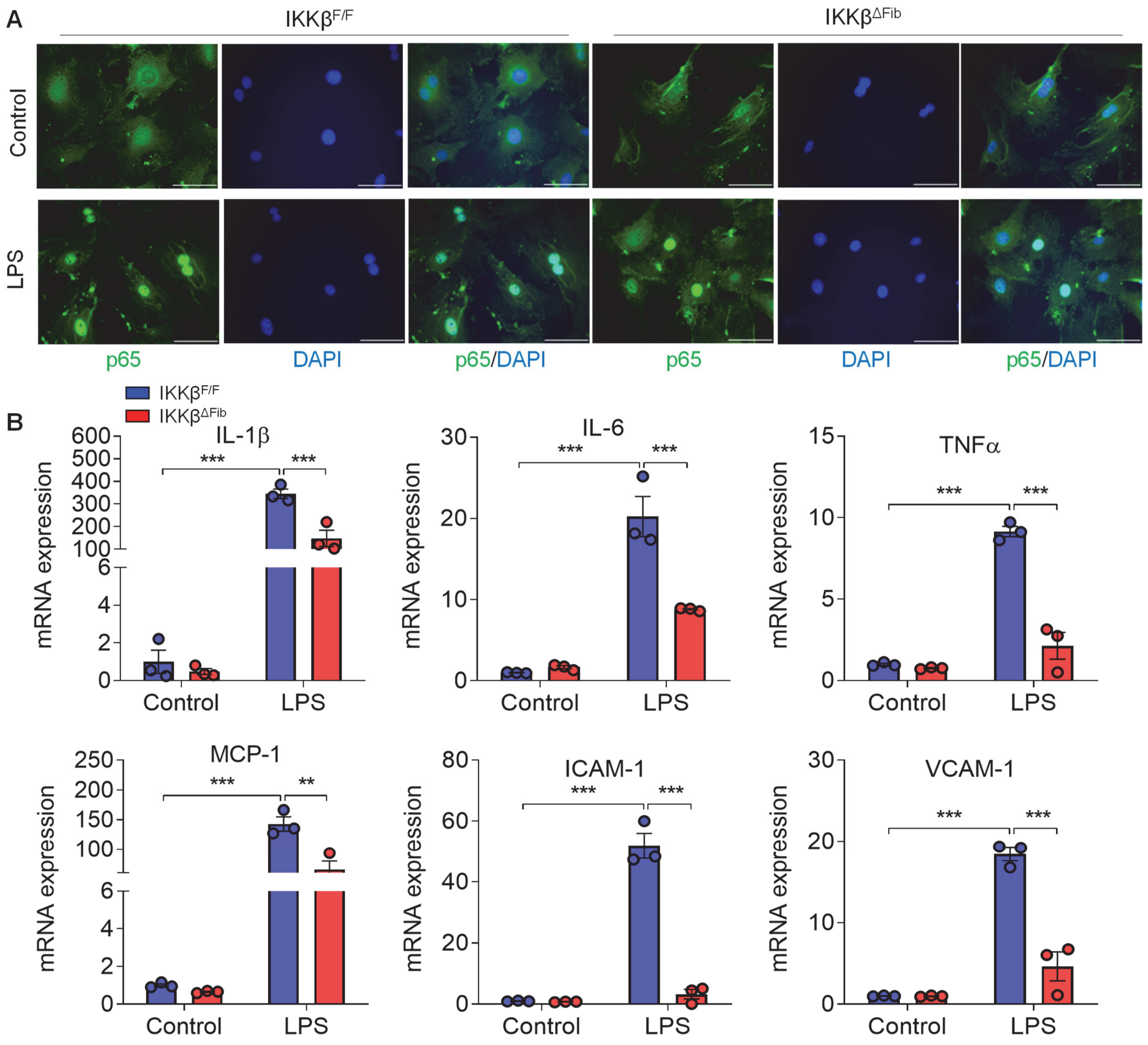

Supplemental Figure 2. Deficiency of IKKβ in cardiac fibroblasts attenuates lipopolysaccharide-induced NF-κB activation and inflammation.

(A) Representative immunofluorescent staining of NF-κB p65 subunit in cardiac fibroblasts isolated from IKKβ$^{F/F}$ and IKKβ$^{\Delta Fib}$ mice that treated with 100 ng/ml of lipopolysaccharide (LPS) or vehicle control for 1 hr (Scale bar, 100μm). (B) QPCR analysis of the mRNA levels of proinflammatory cytokines and adhesion molecules (n=3. Two-way ANOVA ** $P < 0.01$ and *** $P < 0.001$).

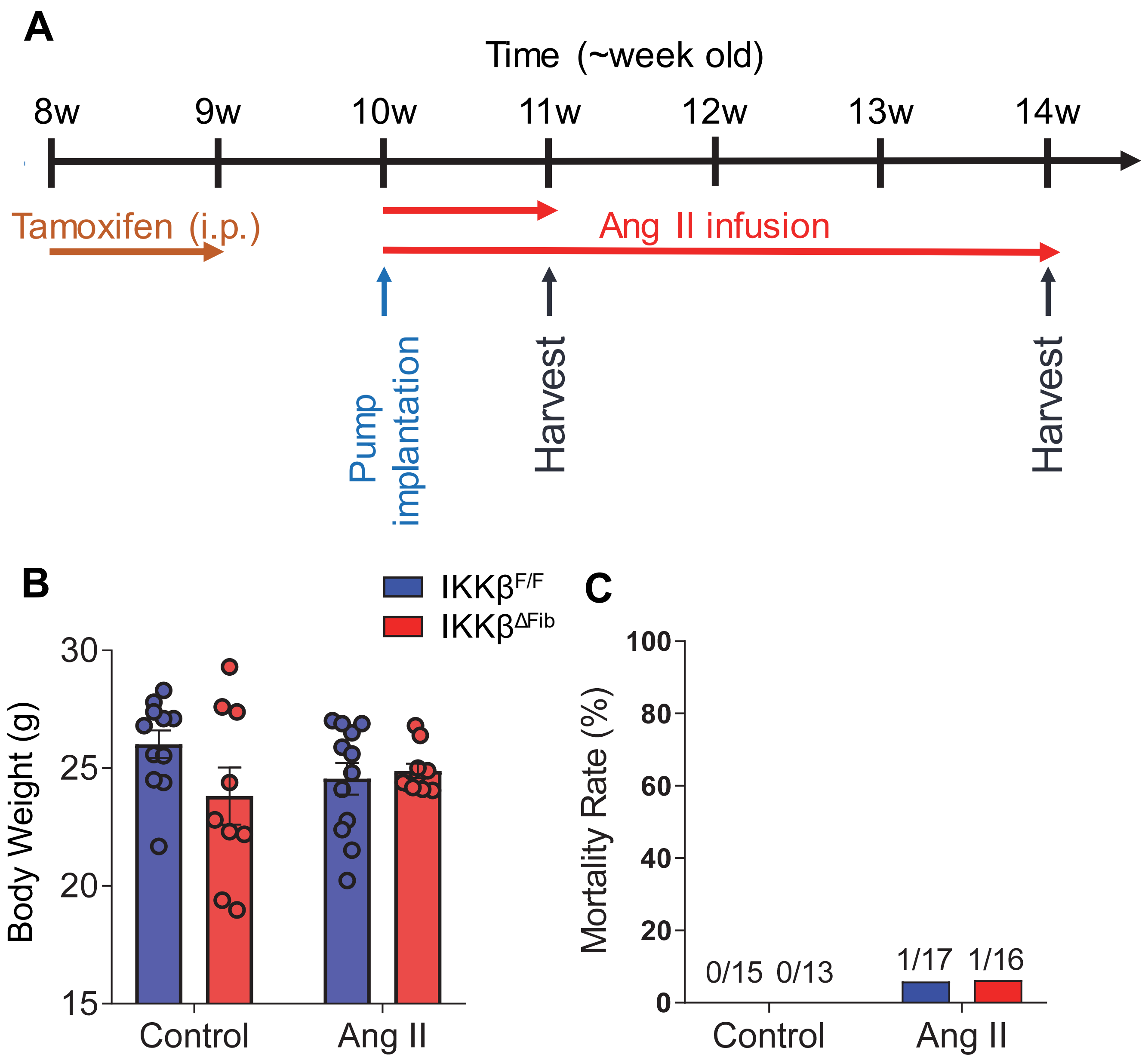

Supplemental Figure 3. **Angiotensin II-induced mouse model of cardiac remodeling.**
(A) Experimental scheme of mice subjected to 1000 ng/kg/min of angiotensin II (Ang II) or control (saline) infusion for 1 and 4 weeks. Eight-week-old male mice were intraperitoneally injected with tamoxifen (2 mg/per day) for 5 consecutive days to induce CreER^{T}-mediated recombination. At the age of 10 weeks, the mice were implanted with mini-osmotic pumps. (B and C) Body weight and mortality rate of male IKKβ$^{F/F}$ and IKKβ$^{\Delta Fib}$ mice after 4-week Ang II or control infusion.

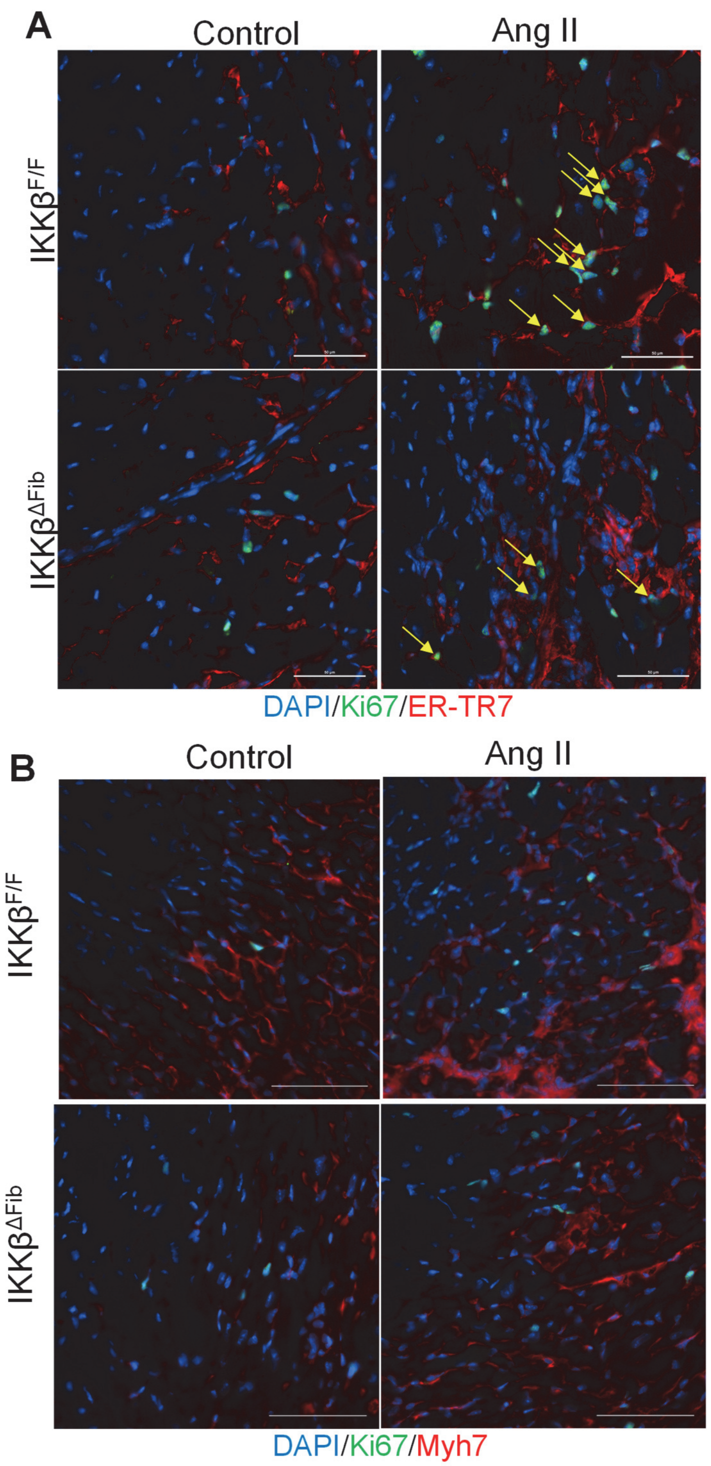

Supplemental Figure 4. Fibroblast IKKβ deficiency reduces angiotensin II-induced cardiac fibroblast proliferation.

Eight-week-old male IKKβ$^{F/F}$ and IKKβ$^{\Delta Fib}$ mice were intraperitoneally injected with 2 mg tamoxifen per day for 5 days. At the age of ten weeks, those mice were infused with 1000 ng/kg/min of angiotensin II (Ang II) or vehicle control for 1 week. Representative images of immunofluorescence staining of Ki-67 and fibroblast cell marker (A) or cardiomyocyte marker (B) in the hearts of IKKβ$^{F/F}$ and IKKβ$^{\Delta Fib}$ mice (Scale bar, A, 50 μm; B, 100 μm).

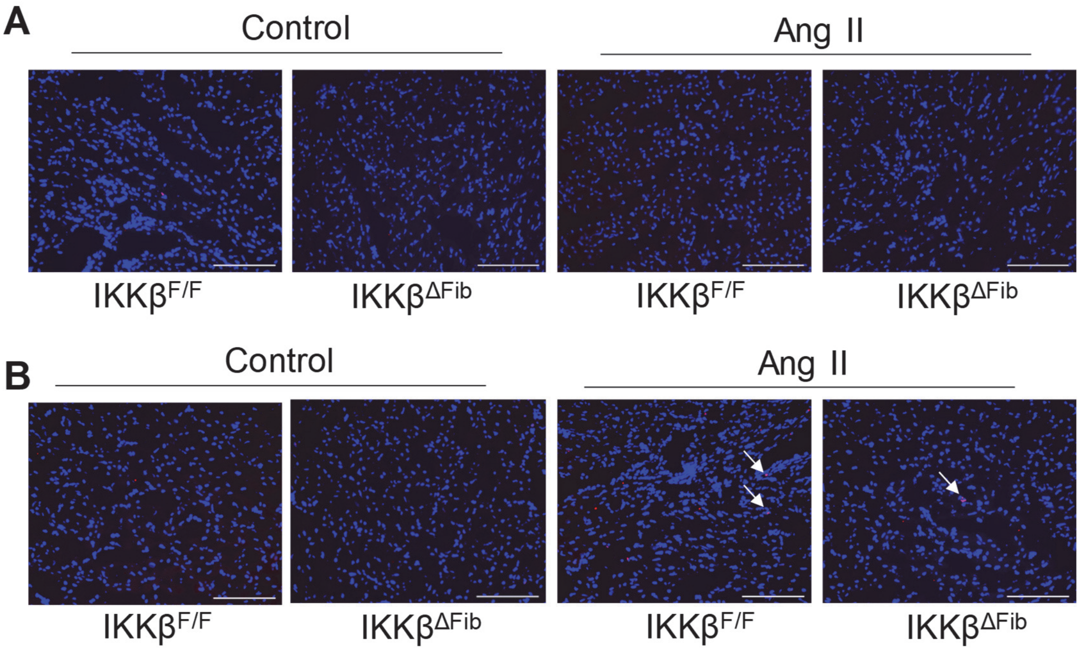

Supplemental Figure 5. Deficiency of fibroblast IKKβ does not increase cell apoptosis in the heart of angiotensin II-infused mice. (A-B) Eight-week-old male IKKβ$^{F/F}$ and IKKβ$^{\Delta Fib}$ mice were intraperitoneally injected with 2 mg tamoxifen per day for 5 days. At the age of ten weeks, the mice were infused with 1000 ng/kg/min of angiotensin II (Ang II) or vehicle control for 1 week (A) or 4 weeks (B). Representative TUNEL staining of hearts from IKKβ$^{\Delta Fib}$ and IKKβ$^{F/F}$ mice. Apoptotic nuclei fluoresce red. The nuclei were visualized with DAPI (blue), and the TUNEL-positive cells were indicated by arrows (Scale bar, 100 μm).

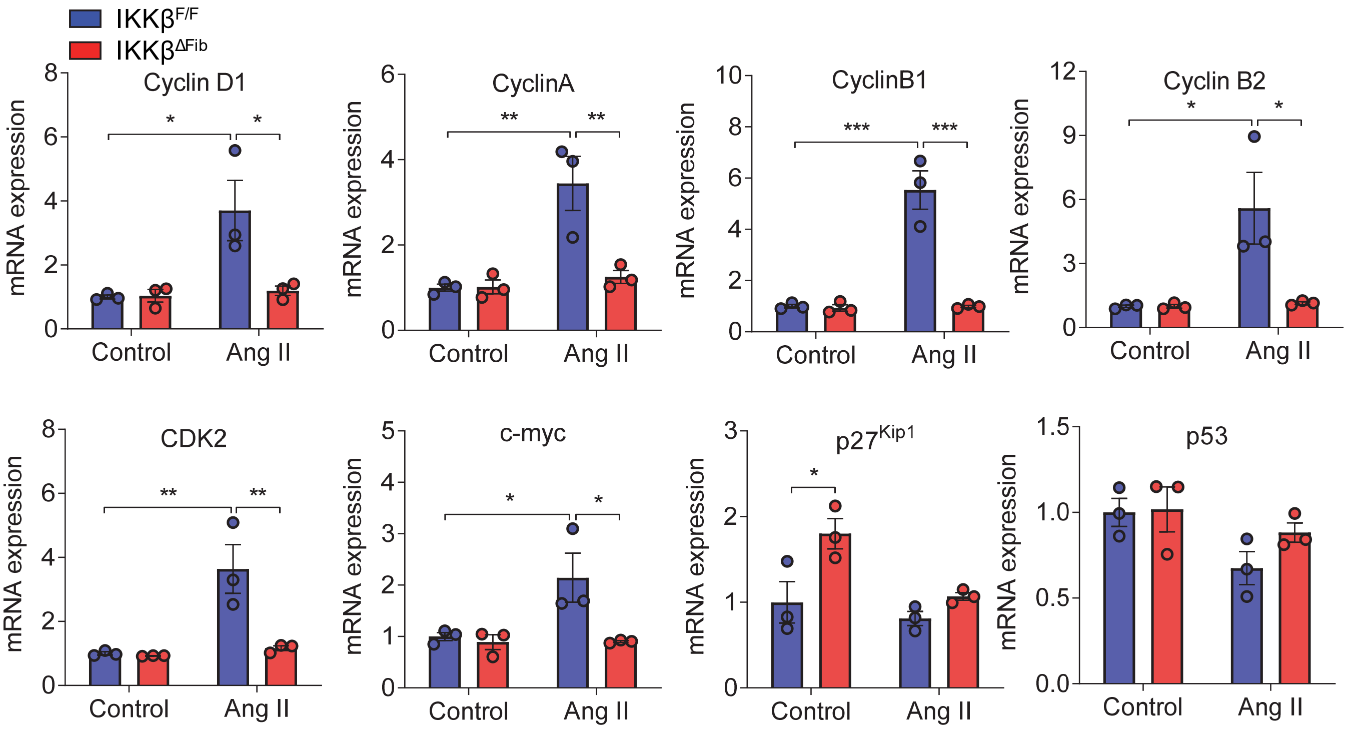

Supplemental Figure 6. Deficiency of IKKβ abolishes the impact of angiotensin II on proliferation or apoptosis-related gene expression in cardiac fibroblasts.

Cardiac fibroblasts were isolated from male IKKβ$^{F/F}$ and IKKβ$^{\Delta Fib}$ mice, and then stimulated with 10^{-6} M of angiotensin II (Ang II) or vehicle control for 24 hr. The expression levels of genes related to proliferation and apoptosis were analyzed by QPCR (n=3, Two-way ANOVA, *$P < 0.05$, **$P < 0.01$, and ***$P < 0.001$).

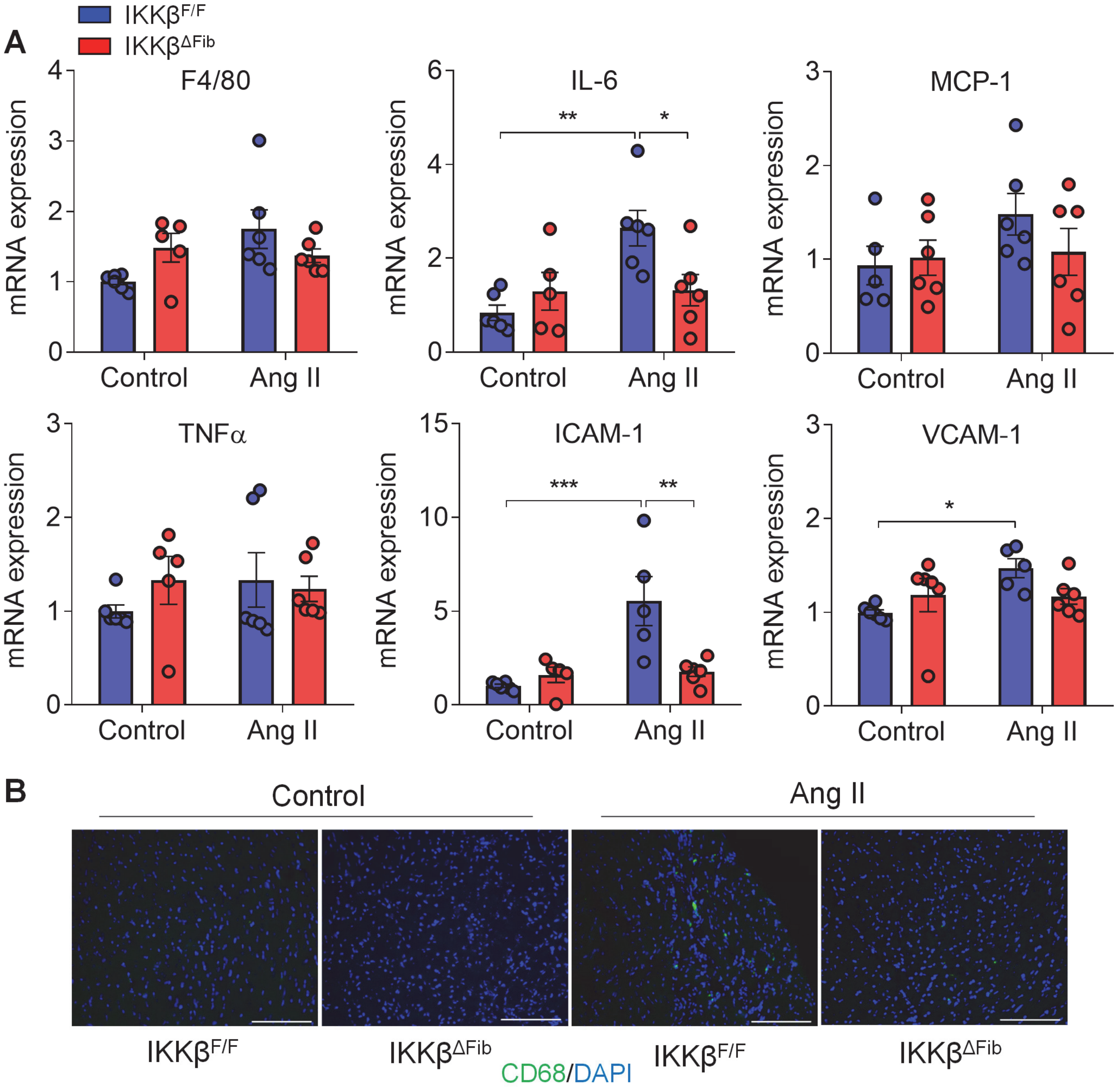

Supplemental Figure 7. Fibroblast IKKβ deficiency modestly affects cardiac inflammation after 4 weeks of angiotensin II infusion.

Eight-week-old male IKKβ$^{F/F}$ and IKKβ$^{\Delta Fib}$ mice were intraperitoneally injected with 2 mg tamoxifen per day for 5 days. At the age of ten weeks, those mice were infused with angiotensin II (Ang II) at the dose of 1000 ng/kg/min for 4 weeks. (A) QPCR analysis of the mRNA levels of inflammatory cytokines and adhesion molecules in the heart of male IKKβ$^{F/F}$ and IKKβ$^{\Delta Fib}$ mice (n=5-6, Two-way ANOVA, **P* < 0.05, ***P* < 0.01 and ****P* < 0.001). (B) Representative images of immunofluorescence staining of CD68 in the heart of IKKβ$^{F/F}$ and IKKβ$^{\Delta Fib}$ mice (n=3, Scale bar, 100 μm).

Supplemental Table 1. Echocardiographic parameters of male IKKβ $^{F/F}$ or IKKβ $^{\Delta Fib}$ mice after 4-week infusion of angiotensin II

Parameters	Control (Saline)		Ang II (1000 ng/kg/min)	
	IKKβ$^{F/F}$	IKKβ$^{\Delta Fib}$	IKKβ$^{F/F}$	IKKβ$^{\Delta Fib}$
HR (bpm)	359.9±17.55	418.66±13.89	407±16.66	406.1±26.7
IVSd (mm)	0.81±0.03	0.79±0.03	1.05±0.04***	0.89±0.04$^{\#}$
IVSs (mm)	1.38±0.03	1.29±0.05	1.50±0.05	1.51±0.05
LVPWd (mm)	0.62±0.02	0.7±0.02	1.03±0.05***	0.80±0.03$^{\#\#\#}$
LVPWs (mm)	1.09±0.03	1.03±0.03	1.36±0.04***	1.18±0.04$^{\#\#}$
LVIDd (mm)	3.82±0.07	3.86±0.09	4.16±0.06**	3.76±0.12$^{\#}$
LVIDs (mm)	2.56±0.10	2.75±0.08	3.14±0.09***	2.52±0.10$^{\#\#\#}$
EF (%)	66.95±1.22	64.52±2.27	42.73±1.63***	67.31±2.56$^{\#\#\#}$
FS (%)	36.55±0.94	35±1.69	20.84±0.95***	37.08±1.91$^{\#\#\#}$

$^{**}P < 0.01$, $^{***}P < 0.001$, compared to IKKβ$^{F/F}$ mice infused with control; $^{\#}P < 0.05$, $^{\#\#}P < 0.01$, $^{\#\#\#}P < 0.001$, compared to IKKβ$^{F/F}$ mice infused with Ang II (n=8-12).

Ang II, angiotensin II; HR, heart rate; IVSd, Inter-ventricular septum diameter at end-Diastole; IVSs, Inter-ventricular septum diameter at end-Systole; LVPWD, Left Ventricular Posterior Wall at end-Diastole; LVPWS, Left Ventricular Posterior Wall at end-Systole; LVIDD, Left Ventricular Internal Dimension at end-Diastole; LVIDS, Left Ventricular Internal Dimension at end-Systole; EF, ejection fraction; FS, Fractional Shortening.

Supplemental Table 2 Primer sequences for quantitative PCR.

Name	Sequence		Name	Sequence	
IKKβ	Forward	5’-GAGCTCAGCCCAAAGAACAG-3’	CTGF	Forward	5’-GGGCCTCTTCTGCGATTTC-3’
	Reverse	5’-AGGTTCTGCATCCCCTCTGG-3’		Reverse	5’-ATCCAGGCAAGTGCATTGGTA-3’
CD68	Forward	5’-CTTCCCACAGGCAGCACAG-3’	CCR2	Forward	5’- GGGTCATGATCCCTATGTGG-3’
	Reverse	5’-AATGATGAGAGGCAGCAAGAGG-3’		Reverse	5’- TCCATGAGCAGTGGTTTGAA-3’
F4/80	Forward	5’-CTTTGGCTATGGGCTTCCAGTC-3’	CXCR2	Forward	5’- ATGCCCTCTATTCTGCCAGAT-3’
	Reverse	5’-GCAAGGAGGACAGAGTTTATCGTG-3’		Reverse	5’- GTGCTCCGGTTGTATAAGATGAC-3’
IL-6	Forward	5’-TAGTCCTTCCTACCCCAATTTCC-3’	CCL4	Forward	5’- TCCCACTTCCTGCTGTTTCTCT -3’
	Reverse	5’-TTGGTCCTTAGCCACTCCTTC-3’		Reverse	5’- CCGTCCTTATCGTAGTCAG-3’
MCP-1	Forward	5’-TTAAAAACCTGGATCGGAACCAA-3’	CDK2	Forward	5’- CTCGACACTGAGACTGAAGGT-3’
	Reverse	5’-GCATTAGCTTCAGATTTACGGGT-3’		Reverse	5’- GCAGCTTGACGATATTAGGGTGA-3’
TNFα	Forward	5’-CCCATATACCTGGGAGGAGTCTTC-3’	Cyclin D1	Forward	5’- ATGCAAGGCCTGAACCTG-3’
	Reverse	5’-CATTCCCTTCACAGAGCAATGAC-3’		Reverse	5’- TCCTCCTCAGTGGCCTTG-3’
IL-1β	Forward	5’-GCAACTGTTCCTGAACTCAACT-3’	c-myc	Forward	5’- CCACCAGCAGCGACTCTGA -3’
	Reverse	5’-ATCTTTTGGGGTCCGTCAACT-3’		Reverse	5’- TGCCTCTTCTCCACAGACACC -3’
ICAM-1	Forward	5’-GTGATCCCTGGGCCTGGTG-3’	Cyclin A	Forward	5’- GATACCTGCTCGGGGAAAGAG -3’
	Reverse	5’-GGAAACGAATACACGGTGATGG-3’		Reverse	5’- GCATTGGGGAAACTGTGTTGA -3’
VCAM-1	Forward	5’-TACCAGCTCCCAAAATCCTG -3’	Cyclin B1	Forward	5’- GCGTGTGCCTGTGACAGTTA-3’
	Reverse	5’-TCTGCTAATTCCAGCCTCGT-3’		Reverse	5’- CCTAGCGTTTTTGCTTCCCTT-3’
GAPDH	Forward	5’-AACTTTGGCATTGTGGAAGG-3’	Cyclin B2	Forward	5’- AGCTCCCAAGGATCGTCCTC-3’
	Reverse	5’-GGATGCAGGGATGATGTTCT-3’		Reverse	5’- TGTCCTCGTTATCTATGTCCTCG-3’
α-SMA	Forward	5’-TCCTGACGCTGAAGTATCCGATA-3’	P21	Forward	5’- ACCCCATACTTCCCCTTCTG -3’
	Reverse	5’-GGCCACACGAAGCTCGTTAT-3’		Reverse	5’- ACCCTAGACCCACAATGCAG-3’
TGF β	Forward	5’-CAAGGGCTACCATGCCAACT-3’	P27	Forward	5’- TGGCTCTGCTCCATTTGACTGTCTG-3’
	Reverse	5’-GTACTGTGTGTCCAGGCTCCAA-3’		Reverse	5’- CTCACGTTTGACATCTTCCTCCTCG-3’
Col1a1	Forward	5’-ATCCTGCCGATGTCGCTAT-3’	Bid	Forward	5’- TCCACAACATTGCCAGACTA -3’
	Reverse	5’-CCACAAGCGTGCTGTAGGT-3’		Reverse	5’- CACTCAAGCTGAACGCAGAG -3’
Col3a1	Forward	5’-CATGACTGTCCCACGTAAGCA-3’	BNIP3	Forward	5’- TCCTGGGTAGAACTGCACTTC -3’
	Reverse	5’-ATTCGCCTTCATTTGATCCCA-3’		Reverse	5’- GCTGGGCATCCAACAGTATTT -3’
Periostin	Forward	5’-CCTGCCCTTATATGCTCTGCT-3’	Gadd45β	Forward	5’- CAACGCGGTTCAGAAGATGC -3’
	Reverse	5’-AAACATGGTCAATAGGCATCACT-3’		Reverse	5’- GGTCCACATTCATCAGTTTGGC-3’
p53	Forward	5’- CCTCTGAGCCAGGAGACATTTTC -3’	NLRP3	Forward	5’- ATTACCCGCCCGAGAAAGG-3’
	Reverse	5’- AAGCCCAGGTGGAAGCCATAGTTG -3’		Reverse	5’- TCGCAGCAAAGATCCACACAG-3’
